# Chinese Herbal Medicine for the Treatment of Children and Adolescents With Refractory *Mycoplasma Pneumoniae* Pneumonia: A Systematic Review and a Meta-Analysis

**DOI:** 10.3389/fphar.2021.678631

**Published:** 2021-06-10

**Authors:** Xiaoying Ling, Xun Sun, Huimin Kong, Shanshan Peng, Zheng Yu, Jiali Wen, Bin Yuan

**Affiliations:** Department of Pediatrics, Affiliated Hospital of Nanjing University of Chinese Medicine, Nanjing, China

**Keywords:** refractory *Mycoplasma pneumoniae* pneumonia, Chinese herbal medicine, complementary and alternative medicine, meta-analysis, effectiveness

## Abstract

**Objectives:** Chinese herb medicine (CHM) is one of the most popular complementary and alternative therapies, which has been widely used to treat Refractory *Mycoplasma Pneumoniae* Pneumonia (RMPP). However, the effect and safety of CHM remain controversial. Hence, we conducted this meta-analysis to evaluate whether CHM combination therapy could bring benefits to children and adolescents with RMPP.

**Methods:** Seven databases were used for data searching through November 11, 2020 following the PRISMA checklist generally. Review Manager 5.3, Trial sequential analysis 0.9.5.10 Beta software and Stata16.0 were applied to perform data analyses. Mean difference or risk ratio was adopted to express the results, where a 95% confidence interval (CI) was applied.

**Results:** In general, this research enrolled 17 trials with 1,451 participants. The overall pooled results indicated that CHM was beneficial for children and adolescents with RMPP by improving the clinical efficacy rate [RR = 1.20, 95% CI (1.15, 1.25), *p* < 0.00001], shortening antipyretic time [MD = −2.60, 95% CI (−3.06, −2.13), *p* < 0.00001], cough disappearance time [MD = −2.77, 95% CI (−3.12, −2.42), *p* < 0.00001], lung rale disappearance time [MD = −2.65, 95% CI (−3.15, −2.15), *p* < 0.00001], lung X-ray infiltrates disappearance time [MD = −2.75, 95% CI (−3.33, −2.17), *p* < 0.00001], reducing TNF-α level [MD = −5.49, 95% CI (−7.21, −3.77), *p* < 0.00001]. Moreover, subgroup results suggested that removing heat-phlegm and toxicity therapy had more advantages in shortening antipyretic time, cough disappearance time, lung X-ray infiltrates disappearance time and reducing TNF-α level. Meanwhile promoting blood circulation therapy seemed to be better at relieving lung rale. However, regarding adverse events, the two groups displayed no statistical difference [RR = 0.97, 95% CI (0.60, 1.57), *p* = 0.91].

**Conclusion:** Despite of the apparently positive results in relieving clinical symptoms, physical signs and reducing inflammation, it is premature to confirm the efficacy of CHM in treating RMPP because of the limitation of quality and the number of the included studies. More large-scale, double-blind, well-designed, randomized controlled trials are needed in future research.

## Introduction

As a significant pathogen, *Mycoplasma pneumoniae* (*M. pneumoniae*) corresponds to 10–40% of community-acquired pneumonia (CAP) in children and adolescence (Waites et al., 2017; Gao et al., 2019; Kurkela et al., 2019). Most children and adolescents have mild symptoms and *M. pneumoniae* infection is perceived to be self-limited. However, after being treated by macrolide antibiotics for no less than 7 days, some children and adolescents still show severe symptoms and/or progressively worsening radiological findings, which can be diagnosed as refractory *M. pneumoniae* pneumonia (RMPP) (Chen et al., 2015). Compared to common *M. pneumoniae* pneumonia (MPP), RMPP commonly presented with persistent or repeated high fever, severe cough, multiple complications involving atelectasis, pleural effusion, myocarditis, hemolytic anemia, encephalitis, and even multiple organ dysfunction, damaging the health of children and adolescents (Zhai et al., 2020). In recent years, the incidence of RMPP has gradually increased, especially in Asia (Guo et al., 2020; Lee et al., 2020), which is becoming a critical issue for pediatricians.

The pathogenesis of RMPP is multifactorial, including macrolide resistance (Zhao et al., 2020), mixed infection (Zhou et al., 2020), excessive inflammatory reaction (Li et al., 2019a), immunity dysfunction (Yu et al., 2017), and blood hypercoagulability (Huang et al., 2021). The first line antibiotics used for RMPP are macrolides. However, high presence of macrolide resistance has been reported in RMPP (Zhang et al., 2018; Zhao et al., 2020). Although tetracyclines and fluoroquinolones are second-line antibiotics, side effects limit their use in children (Tsai et al., 2020). Corticosteroids can reduce inflammatory responses and have been confirmed to be an effective treatment for RMPP (Kim et al., 2019), but its resistance has been reported in some RMPP cases with more severe presentations (Yan et al., 2016). Intravenous immunoglobulin (IVIG) is another therapy, but the uniform standard for the starting time, dose, and duration has not been established (Yang et al., 2017). Hence, the exploration of an effective and safe pharmacological strategy for children and adolescents with RMPP is highly significant.

Chinese herb medicine (CHM) is a prevailing alternative and complementary therapy (Wang, 2020), and previous reviews have demonstrated that CHM displayed obvious advantages in MPP (Li et al., 2019c; Zhang et al., 2020). In our study, we aimed to focus on the role of CHM in RMPP, as the condition is more critical and treatment is more difficult. Traditional Chinese Medicine (TCM) theory classifies RMPP into the category of “pneumonia with dyspneic cough”. The key pathogenesis of RMPP is asthenia in origin and excess in superficiality. Asthenia in origin include Qi deficiency and Yin deficiency. Excess in superficiality include phlegm heat, pathogenic toxin, and blood stasis. Based on the theory above, classic TCM therapies involves removing heat-phlegm and toxicity, promoting blood circulation, invigorating Qi and consolidating superficies, supplementing Qi and nourishing Yin (Liu and Ma, 2017; Yan et al., 2019). As we all know, CHM has the characteristics of multiple components, targets and pathways, including inhibiting *M. pneumoniae*, modulating immunity, repairing damaged epithelial cells, improving microcirculation, and alleviating adverse effects of Western medicine (WM) (Tan and Jiang, 2018). For instance, Yupingfeng granule, a kind of Chinese medicine prescription, could reduce inflammation and regulate immune function to benefit RMPP children and adolescents (Wang et al., 2018). Zhou reported the therapy of promoting blood circulation could ameliorate symptoms of children and adolescents with RMPP by anti-inflammatory, improving lung microcirculation and promoting tissue repair (Zhou, 2018). Therefore, we conducted this meta-analysis with the aim to evaluate the effect and safety of CHM and compare the clinical efficacy of different TCM therapies in RMPP.

## Materials and Methods

The Preferred Reporting Items for Systematic Reviews and Meta-Analysis (PRISMA) guidelines (Moher et al., 2015) were followed to perform this systematic review, which was registered in PROSPERO (CRD42020218609). A completed PRISMA checklist was exhibited in Supplementary File S1.

### Literature Search Strategy

Seven databases, such as PubMed, embase, Cochrane Library, China National Knowledge Infrastructure database (CNKI), VIP database for Chinese Technical Periodicals (VIP), Wanfang Data and China Biomedical Medicine database (CBM), were searched from inception to November 11, 2020. Search terms were used as follow: “refractory *mycoplasma pneumoniae* pneumonia”, “RMPP”, “traditional Chinese medicine”, “children”, “adolescents”, “randomized controlled trial” etc. Details of all databases of search strategies were shown in Supplementary Table S1. Reference lists and conference proceedings were also checked manually for additional relevant data. We contacted relevant corresponding authors for missing or unreported information when needed. No restrictions were applied on language or publication date.

### Inclusion Criteria

Studies meeting the following conditions were included for further analysis: 1) Study design: prospective, randomized controlled trials (RCTs); 2) Participants: all the enrolled children and adolescents within 18 years old were required to meet any proper diagnostic criteria of RMPP, such as “Expert consensus on the diagnosis and treatment of *Mycoplasma pneumoniae* pneumonia in children” (Chen et al., 2015); 3) Interventions and Comparisons: patients receiving CHM (no restriction on dosage, formula, and form) combined with WM in intervention groups, while WM alone as the control; 4) Outcomes: at least one primary outcome of interest with reliable and available data.

### Exclusion Criteria

Cases meeting the following conditions were excluded: 1) case-control or cohort trials, quasi-randomized studies; 2) cell or animal experiments, case reports, meta-analyses, reviews; 3) studies with poor design, unavailable data, or unreported target outcomes; 4) children and adolescents who received other joint interventions such as acupuncture, cupping, moxibustion, massage, acupoint application.

### Outcome Measures

The primary outcomes were clinical efficacy rate, antipyretic time, and cough disappearance time. The secondary outcomes included the disappearance time of lung rale and lung X-ray infiltrates, inflammation index and adverse effects.

### Data Extraction and Quality Assessment

From each study, the collected data are: 1) study characteristics, including publication year, location, as well as the name of the first author; 2) participants information, such as age, gender, sample size, course of disease, and diagnosis standards; 3) details of interventions, including treatment protocols, TCM therapies, and duration; 4) outcome measures.

The Cochrane risk of bias tool (Higgins et al., 2011) was adopted to the evaluation of the selected studies’ methodological quality based on considerations below: random sequence generation, allocation concealment, blinding of participants and personnel, blinding of outcome assessment, incomplete outcome data, selective reporting, and other bias. The bias risk assessment was independently conducted by two investigators with three potential responses: low, high and unclear. A third author was also invited to resolve any conflict.

### Data Synthesis and Statistical Analysis

Review Manager 5.3 was applied for statistical analysis. The risk ratio (RR) with 95% confidence intervals (CI) was used to express the results of dichotomous data. Mean difference (MD) or standardized mean difference (SMD) with a 95% CI was adopted for continuous data. Heterogeneous results could not be avoided due to the use of different herbal medicines in all selected studies. For this reason, summary effect measures were calculated by the random-effects method. Statistical heterogeneity was quantified through the I-squared (*I* ^*2*^) statistic, whereby an *I* ^*2*^ > 50% was deemed substantial heterogeneity (Higgins et al., 2003). Subgroups analyses were performed and categorized according to types of WM and different TCM therapies described in each study, respectively, which could not only reduce heterogeneity to some extent but also compare the clinical efficacy of different TCM therapies in RMPP. The differences at a statistically significant level were defined by *p* < 0.05. Publication bias was assessed through a funnel plot and calculated by Egger’s tests, when at least ten studies were available. Sensitivity analyses were conducted by changing the random-effects method to a fixed-effect model and deleting each study in sequence to evaluate the stability of the results and explore the sources of heterogeneity.

Some positive findings from meta-analysis might be caused by a random error (by chance) rather than the true effects of the intervention and these results might often increase the likelihood of overestimation (Type I errors) or underestimation (Type II errors) (Pereira and Ioannidis, 2011; Afshari et al., 2017), which could be resolved by Trial sequential analysis (TSA) (Kang, 2021). Therefore, we performed TSA by using TSA 0.9.5.10 Beta software to reduce the false-positive results caused by random errors and determine whether more RCTs were needed to obtain robust conclusions. The error rate was set as 5% for type I and 10% for type II.

## Results

### Search Results and Study Characteristics

**FIGURE 1:**
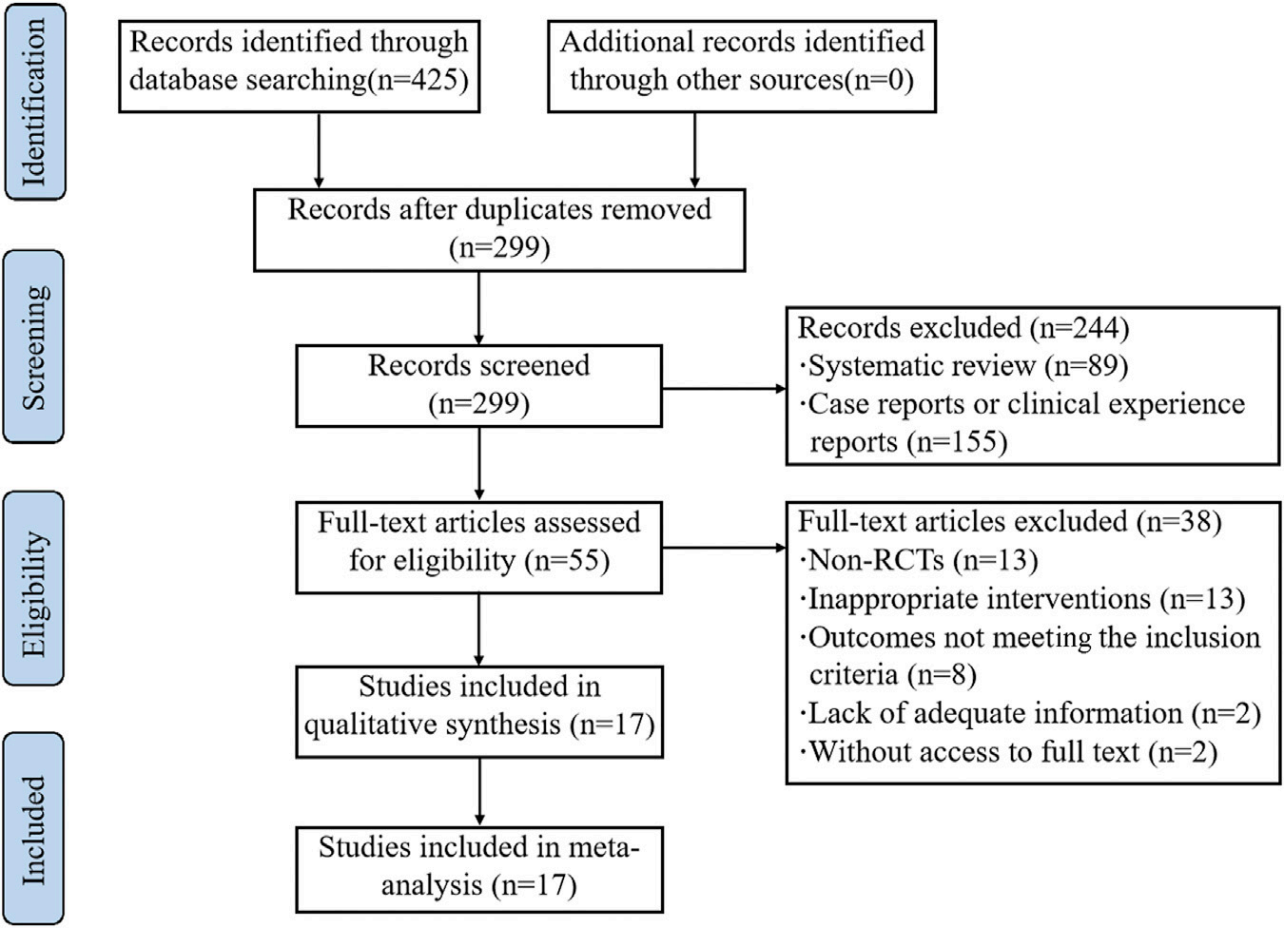
PRISMA flow diagram of the literature search process.

A total of 425 articles were retrieved with the predefined search strategy. 299 studies remained after we removed duplicates. By screening titles and abstracts, 244 articles were excluded, and 55 articles were yielded to conduct a full-text evaluation. Among them, 38 articles were excluded, which included non-RCTs (*n* = 13); trials with inappropriate interventions (*n* = 13); trials in which outcomes did not meet the inclusion criteria (*n* = 8); studies lack of adequate drug information (*n* = 2); articles without access to full text (*n* = 2). Finally, 17 RCTs (Qian, 2011; Chen, 2014; Meng, 2015; Tian, 2016; Wang and Wu, 2016; Yuan et al., 2016; Lian, 2017; Sun et al., 2017; Tan and Yang, 2017; Zhong et al., 2017; Wang et al., 2018; Wang, 2018; Yang, 2019; He et al., 2020a; He et al., 2020b; Zhang, 2020a; Zhang, 2020b) were eligible for meta-analysis (Figure 1). A list of excluded studies by reading full-text was provided in Supplementary File S2.

All studies were single-center and parallel-design RCTs conducted in China between 2009 and 2019. A total of 1,451 participants were recruited, with 726 in intervention groups and 725 in control groups. WM in our analysis could be divided into two categories: nine studies (Chen, 2014; Meng, 2015; Tian, 2016; Yuan et al., 2016; Sun et al., 2017; Zhong et al., 2017; Wang et al., 2018; He et al., 2020a; Zhang, 2020b) using macrolide antibiotics, and the other eight (Qian, 2011; Wang and Wu, 2016; Lian, 2017; Tan and Yang, 2017; Wang, 2018; Yang, 2019; He et al., 2020b; Zhang, 2020a) using macrolide antibiotics joint with corticosteroids. Ten studies (Qian, 2011; Chen, 2014; Tian, 2016; Yuan et al., 2016; Lian, 2017; Wang, 2018; Yang, 2019; He et al., 2020a; He et al., 2020b; Zhang, 2020a) administrated CHM to participants in decoction form, three (Wang and Wu, 2016; Sun et al., 2017; Wang et al., 2018) in granule, three (Meng, 2015; Tan and Yang, 2017; Zhang, 2020b) in injection and one (Zhong et al., 2017) in oral liquid. TCM therapies were described in all studies, nine (Qian, 2011; Yuan et al., 2016; Lian, 2017; Sun et al., 2017; Tan and Yang, 2017; Zhong et al., 2017; He et al., 2020a; He et al., 2020b; Zhang, 2020b) with removing heat-phlegm and toxicity therapy, four (Chen, 2014; Tian, 2016; Wang, 2018; Zhang, 2020a) with promoting blood circulation therapy and the rest (Wang and Wu, 2016; Sun et al., 2017; Wang et al., 2018; Yang, 2019) with invigorating Qi and consolidating superficies therapy. The treatment duration ranged from one week to four weeks. Table 1 listed the characteristics of selected studies. Supplementary Tables S3, S4 provided detailed information on CHM.

**TABLE 1:** Baseline characteristics of included studies.

Study Id	Region	Sample size (T/C)	Age (Y)		Gender (M/F)		Course of disease (days)		Diagnosis standards	TCM therapies	Intervention		Duration (weeks)	Outcomes
			T	C	T	C	T	C			T	C		
Sun et al. (2017)	China	40/40	7.75 ± 2.64	7.86 ± 2.33	22/18	23/17	NR	NR	Practical pediatrics of Zhu Futang	Invigorating Qi and consolidating superficies therapy	MA + Yupingfeng granule. Ages: ≤3 (2.5 g); ＞3 (5 g), tid, po	MA (no details)	2	③④⑤⑦
Meng (2015)	China	50/50	6.5 ± 2.0	6.6 ± 2.1	26/24	25/25	4.0 ± 1.9	3.9 ± 1.8	NR	Removing heat-phlegm and toxicity therapy	MA + Reduning injection. Ages:1–2 (4 ml); 3–5(6 ml); 6–14 (10 ml), qd, ivgtt	MA (erythromycin 20–30 mg/kg, qd, ivgtt)	1	①②③
Zhang (2020b)	China	75/75	8.25 ± 1.56	8.57 ± 1.23	38/37	36/39	4.24 ± 1.15	4.03 ± 1.52	Practical pediatrics of Zhu Futang	Removing heat-phlegm and toxicity therapy	MA + Asarone injection (0.5 ml/kg, qd, ivgtt)	MA (azithromycin 10 mg/kg, qd, po)	2	①③④⑤⑥
Zhang (2020a)	China	30/30	8.24 ± 1.86	7.65 ± 2.36	16/14	NR	NR	NR	Practical pediatrics of Zhu Futang	Promoting blood circulation therapy	MA + CS + Self-made formula (qd, po)	MA (no details)+ CS (2 mg/kg, qd, ivgtt)	2	①②③④
Wang (2018)	China	33/33	7.65 ± 2.58	7.42 ± 2.61	18/15	17/16	NR	NR	Practical pediatrics	Promoting blood circulation therapy	MA + CS + Self-made formula (bid, po)	MA (no details)+ CS (2 mg/kg, qd, po)	2	①②③④⑦
Wang et al. (2018)	China	50/50	7.18 ± 1.24	7.26 ± 1.23	23/27	24/26	6.16 ± 4.20	6.24 ± 4.13	Practical pediatrics of Zhu Futang	Invigorating Qi and consolidating superficies therapy	MA + Yupingfeng granule. Ages: ≤3 (2.5 g); ＞3 (5 g), qd, po	MA (Azithromycin10 mg/kg, qd, po)	2	③④⑤⑥
Wang and Wu (2016)	China	30/32	6.30 ± 2.74	5.78 ± 2.88	19/11	19/13	NR	NR	(Cao (2010))	Invigorating Qi and consolidating superficies therapy	MA + CS + Yupingfeng granule. Ages: ≤3 (2.5 g); ＞3 (5 g), tid, po	MA (no details)+ CS (no details)	4	②⑤
Tian (2016)	China	25/25	6.5 ± 2.4	6.5 ± 2.3	11/14	13/12	4.1 ± 1.7	4.0 ± 1.8	NR	Promoting blood circulation therapy	MA + self-made formula (bid, po)	MA (erythromycin 20–30 mg/kg, qd, ivgtt)	1	①②③
Yuan et al. (2016)	China	42/42	4.7 ± 1.4	4.8 ± 1.7	20/22	21/21	NR	NR	NR	Removing heat-phlegm and toxicity therapy	MA + self-made formula (bid, po)	MA (Azithromycin10 mg/kg, qd, ivgtt for 5 days and po for the rest days)	2	①②③④
Tan and Yang (2017)	China	40/40	6.4 ± 3.1	6.6 ± 3.2	16/24	17/23	24.6 ± 3.8	23.8 ± 4.3	Practical pediatrics of Zhu Futang	Removing heat-phlegm and toxicity therapy	MA + CS + andrographolide injection (5–10 mg/kg, qd, ivgtt)	MA (azithromycin 10 mg/kg, qd, ivgtt)+ CS (5–10 mg/kg, qd, ivgtt)	1	①⑦
He et al. (2020a)	China	40/40	9.36 ± 1.22	9.42 ± 1.21	20/20	21/19	19.21 ± 2.38	19.17 ± 2.34	Guidelines (Li et al. (2013))	Removing heat-phlegm and toxicity therapy	MA + self-made formula (bid, po)	MA (erythromycin, 20 mg/kg, tid, ivgtt and azithromycin, 10 mg/kg, qd, po)	2	①②③④⑦
He et al. (2020b)	China	59/57	8.5 ± 1.3	8.5 ± 1.3	35/24	34/23	14.9 ± 1.3	15.0 ± 1.3	Guidelines (Chen et al. (2015))	Removing heat-phlegm and toxicity therapy	MA + CS + self-made formula (bid, po)	MA (erythromycin 20–40 mg/kg, tid, ivgtt for 5–7 days and po for the rest days)+ CS (1–2 mg/kg, qd, ivgtt for 3–5 days and po for the rest days)	2	①⑦
Lian (2017)	China	36/36	7.25 ± 2.80	6.92 ± 2.56	17/19	13/23	10.89 ± 3.2	10.56 ± 2.5	Practical pediatrics of Zhu Futang	Removing heat-phlegm and toxicity therapy	MA + CS + self-made formula (bid, po)	MA (azithromycin 10 mg/kg, qd, ivgtt for 3 days and po for the rest days)+ CS(1 mg/kg, bid, ivgtt)	2	①②③④⑦
Zhong et al. (2017)	China	46/46	8.64 ± 3.05	8.91 ± 3.13	25/21	28/18	15.19 ± 3.62	14.86 ± 3.58	Practical pediatrics of Zhu Futang	Removing heat-phlegm and toxicity therapy	MA + shedan chuanbei oral liquid (10 ml,bid,po)	MA (azithromycin 10 mg/kg, qd, ivgtt)	2	①②③④⑥
Qian (2011)	China	37/37	2–14	2–14	NR	NR	NR	NR	(Cao (2010))	Removing heat-phlegm and toxicity therapy	MA + CS + Self-made formula (bid, po)	MA (azithromycin 10 mg/kg, qd, ivgtt for 5 days and po for the rest days) + CS(2 mg/kg, qd, ivgtt)	2	①②③④
Yang (2019)	China	33/32	7.33 ± 3.30	8.16 ± 3.60	18/15	18/14	57.79 ± 21.92	54.5 ± 29.48	Guidelines (Chen et al. (2015))	Invigorating Qi and consolidating superficies therapy	MA + CS + Self-made formula (bid, po)	MA (azithromycin 10 mg/kg, qd, po)+ CS(1 mg/kg, qd, po)	3	①⑦
Chen (2014)	China	60/60	1.5 ± 0.3	1.5 ± 0.3	NR	NR	NR	NR	Practical pediatrics of Zhu Futang	Promoting blood circulation therapy	MA + self-made formula (bid, po)	MA (no details)	2	①

T, treatment; C, control; Y, year; M, male; F, female; NR, not reported; MA, macrolide antibiotics; CS, corticosteroids; ① clinical efficacy rate; ② antipyretic time; ③ cough disappearance time; ④ lung rale disappearance time; ⑤ lung X-ray infiltrates disappearing time; ⑥ inflammation index; ⑦ adverse effects.

### Methodological Bias of the Included Studies

**FIGURE 2:**
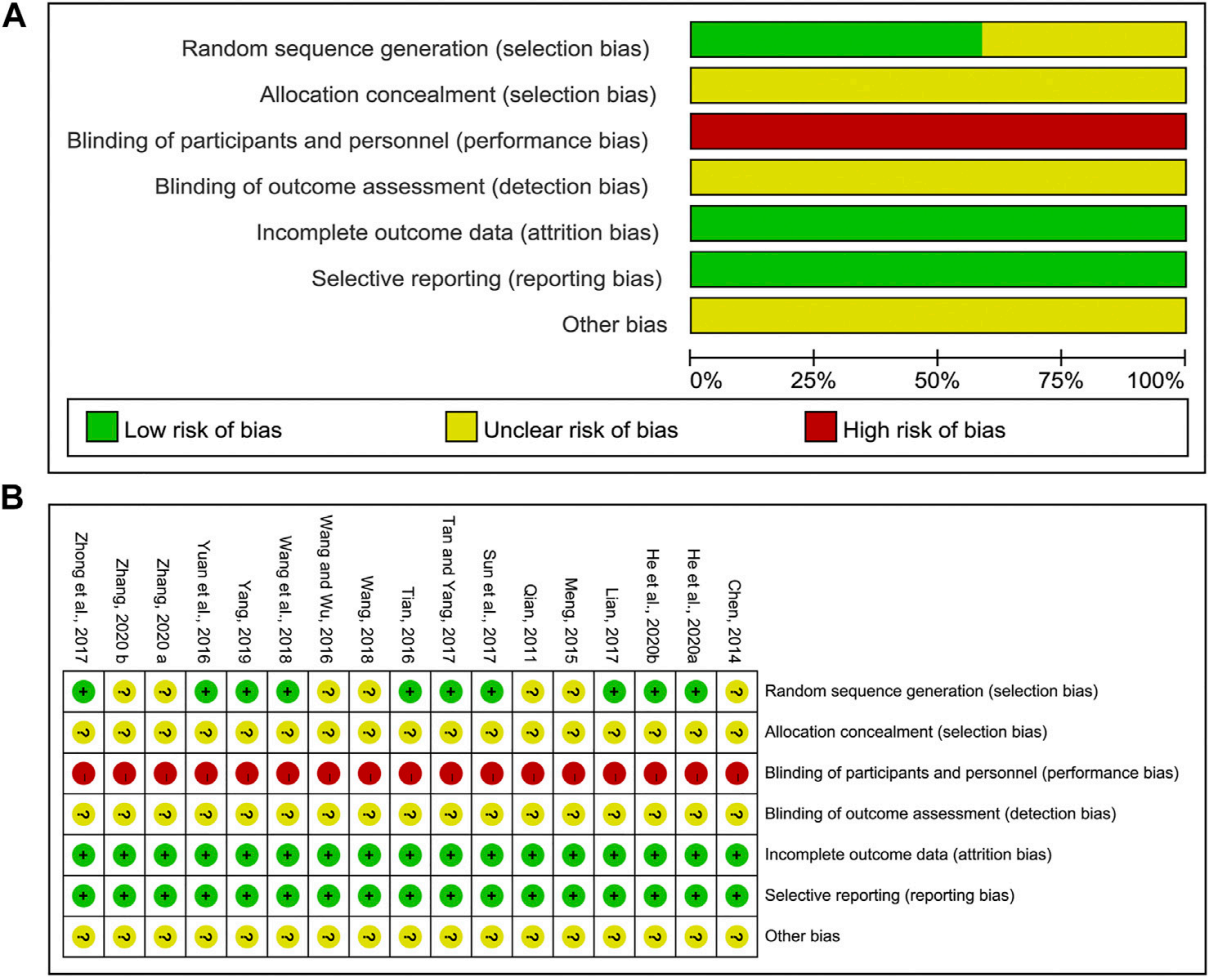
Risk of methodological bias of the included studies. **(A)** Risk of bias graph. **(B)** Risk of bias summary.

The results of methodological bias were presented in Figure 2. All included studies mentioned the randomized allocation. Ten clearly described the randomization method, including random number table (Yuan et al., 2016; Lian, 2017; Sun et al., 2017; Tan and Yang, 2017; Zhong et al., 2017; Wang et al., 2018; Yang, 2019; He et al., 2020a) or computer-generated numbers (Tian, 2016) or ballot (He et al., 2020b), which were considered as the low risk, while seven (Qian, 2011; Chen, 2014; Meng, 2015; Wang and Wu, 2016; Wang, 2018; Zhang, 2020a; Zhang, 2020b) did not provide the detailed randomization methodology. Allocation concealment was unclear and none of them were double-blinded. It was easy for participants to identify which group they were in, since the packages of CHM and WM were different. All articles had complete data and consistent outcomes as described in method section. And other biases were unclear.

### Outcome Measures

#### Clinical Efficacy Rate

**FIGURE 3:**
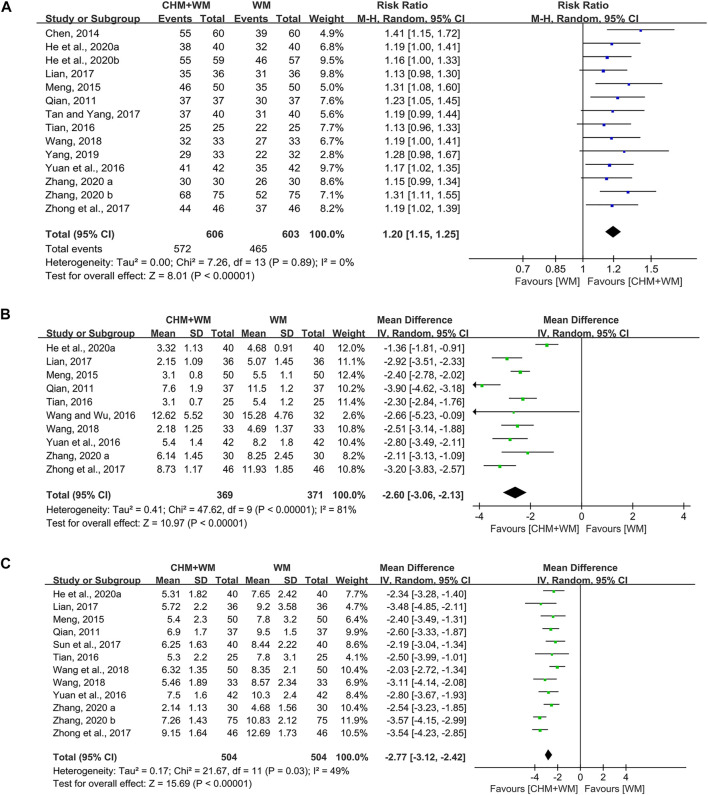
Forest plots of primary outcomes. **(A)** Clinical efficacy rate. **(B)** Antipyretic time. **(C)** Cough disappearance time.

Fourteen studies (1,209 patients) (Qian, 2011; Chen, 2014; Meng, 2015; Tian, 2016; Yuan et al., 2016; Lian, 2017; Tan and Yang, 2017; Zhong et al., 2017; Wang, 2018; Yang, 2019; He et al., 2020a; He et al., 2020b; Zhang, 2020a; Zhang, 2020b) reported clinical efficacy rate. The pooled data indicated that combination of CHM and WM improved clinical efficacy significantly [RR = 1.20, 95% CI (1.15, 1.25), *p* < 0.00001; *I* ^*2*^ = 0%] (Figure 3A). Subgroup analyses (Table 2) showed there was no difference in this result when classified by the type of WM. In terms of TCM differentiation, three therapies obtained the similar clinical efficacy rate.

**TABLE 2:** Summarized results of subgroup analyses.

Outcomes	Subgroup	Trial (n)	Sample size (n)	Effect estimate RR/MD (95%CI)	*I* ^*2*^	*P*
Clinical efficacy rate	Classification of WM					
	MA	8	756	1.22 (1.15, 1.30)	0%	<0.00001
	MA + CS	6	453	1.17 (1.10, 1.25)	0%	<0.00001
	Classification of CHM					
	Removing heat-phlegm and toxicity therapy	9	848	1.20 (1.14, 1.26)	0%	<0.00001
	Promoting blood circulation therapy	4	296	1.20 (1.09, 1.33)	27%	0.0003
	Invigorating Qi and consolidating superficies therapy	1	65	1.28 (0.98, 1.67)	NR	0.07
Antipyretic time	Classification of WM					
	MA	5	406	−2.38 (−2.98, −1.78)	85%	<0.00001
	MA + CS	5	334	−2.89 (−3.52, −2.25)	64%	<0.00001
	Classification of CHM					
	Removing heat-phlegm and toxicity therapy	6	502	−2.73 (−3.42, −2.05)	89%	<0.00001
	Promoting blood circulation therapy	3	176	−2.35 (−2.73, −1.97)	0%	<0.00001
	Invigorating Qi and consolidating superficies therapy	1	62	−2.66 (−5.23, −0.09)	NR	0.04
Cough disappearance time	Classification of WM					
	MA	8	736	−2.72 (−3.22, −2.23)	64%	<0.00001
	MA + CS	4	272	−2.75 (−3.18, −2.32)	0%	<0.00001
	Classification of CHM					
	Removing heat-phlegm and toxicity therapy	7	652	−3.01 (−3.43, −2.58)	44%	<0.00001
	Promoting blood circulation therapy	3	176	−2.69 (−3.22, −2.16)	0%	<0.00001
	Invigorating Qi and consolidating superficies therapy	2	180	−2.09 (−2.63, −1.56)	0%	<0.00001
Lung rale disappearance time	Classification of WM					
	MA	6	586	−2.20 (−2.83, −1.56)	69%	<0.00001
	MA + CS	4	272	−3.25 (−3.65, −2.85)	0%	<0.00001
	Classification of CHM					
	Removing heat-phlegm and toxicity therapy	6	552	−2.83 (−3.34, −2.32)	58%	<0.00001
	Promoting blood circulation therapy	2	126	−3.27 (−3.85, −2.69)	0%	<0.00001
	Invigorating Qi and consolidating superficies therapy	2	180	−1.26 (−1.96, −0.55)	0%	0.0005
Lung X-ray infiltrates disappearance time	Classification of WM					
	MA	3	330	−2.67 (−3.36, −1.98)	13%	<0.00001
	MA + CS	1	62	−3.22 (−6.36, −0.08)	0%	0.04
	Classification of CHM					
	Removing heat-phlegm and toxicity therapy	1	150	−3.02 (−3.72, −2.32)	NR	<0.00001
	Invigorating Qi and consolidating superficies therapy	3	242	−2.15 (−3.20, −1.11)	0%	<0.0001
TNF-α	Classification of WM					
	MA	3	342	−5.49 (−7.21, −3.77)	58%	<0.00001
	Classification of CHM					
	Removing heat-phlegm and toxicity therapy	2	242	−5.94 (−7.92, −3.95)	61%	<0.00001
	Invigorating Qi and consolidating superficies therapy	1	100	−4.22 (−6.73, −1.71)	NR	0.001
Adverse effects	Classification of WM					
	MA	3	240	0.90 (0.38, 2.12)	38%	0.81
	MA + CS	4	319	0.99 (0.49, 2.00)	0%	0.98
	Classification of CHM					
	Removing heat-phlegm and toxicity therapy	4	348	0.86 (0.47, 1.57)	0%	0.62
	Promoting blood circulation therapy	1	66	1.67 (0.43, 6.41)	NR	0.46
	Invigorating Qi and consolidating superficies therapy	2	145	0.98 (0.34, 2.87)	17%	0.98

RR, risk ratio; MD, mean difference; CI, confidence interval; CHM, Chinese herb medicine; WM, Western medicine; MA, macrolide antibiotics; CS, corticosteroids.

### Antipyretic Time

Ten studies (740 patients) (Qian, 2011; Meng, 2015; Tian, 2016; Wang and Wu, 2016; Yuan et al., 2016; Lian, 2017; Zhong et al., 2017; Wang, 2018; He et al., 2020a; Zhang, 2020a) mentioned antipyretic time. Overall analyses revealed that CHM combinated with WM showed more advantages in shortening antipyretic time than WM alone [MD = −2.60, 95% CI (−3.06, −2.13), *p* < 0.00001, *I* ^*2*^ = 81%] (Figure 3B). In subgroup analyses (Table 2), we found that this outcome might be associated with TCM therapy, as removing heat-phlegm and toxicity therapy brought more benefits compared with promoting blood circulation therapy and invigorating Qi and consolidating superficies therapy.

### Cough Disappearance Time

Twelve studies (1,008 patients) (Qian, 2011; Meng, 2015; Tian, 2016; Yuan et al., 2016; Lian, 2017; Sun et al., 2017; Zhong et al., 2017; Wang, 2018; Wang et al., 2018; He et al., 2020a; Zhang, 2020a; Zhang, 2020b) were identified. A significant difference was found between groups, since the cough disappearance time in children and adolescents treated with CHM and WM was shorter than those treated with WM alone [MD = −2.77, 95% CI (−3.12, −2.42), *p* < 0.00001, *I* ^*2*^ = 49%] (Figure 3C). Results of subgroup analyses (Table 2) yielded that removing heat-phlegm and toxicity therapy might work faster than the two others.

### Lung Rale Disappearance Time

**FIGURE 4:**
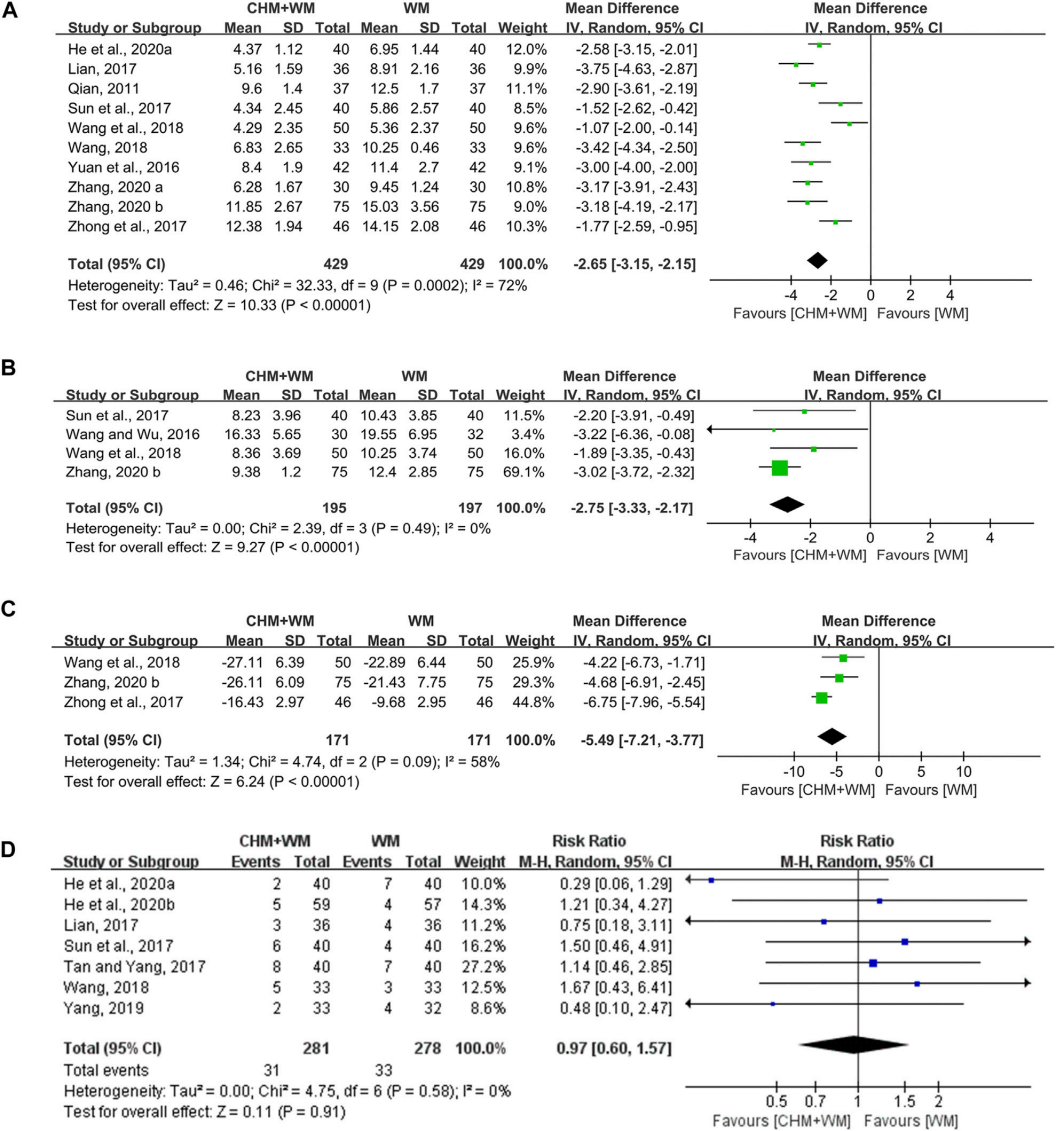
Forest plots of secondary outcomes. **(A)** Lung rale disappearance time. **(B)** Lung X-ray infiltrates disappearance time. **(C)** TNF-α. **(D)** Adverse effects.

Ten studies (858 patients) (Qian, 2011; Yuan et al., 2016; Lian, 2017; Sun et al., 2017; Zhong et al., 2017; Wang, 2018; Wang et al., 2018; He et al., 2020a; Zhang, 2020a; Zhang, 2020b) evaluated this outcome measure. Meta-analysis showed that intervention with CHM and WM resulted in a greater decrease than WM alone [MD = −2.65, 95% CI (−3.15, −2.15), *p* < 0.00001, *I* ^*2*^ = 72%] (Figure 4A). Categorized by TCM therapy, the subgroup analyses (Table 2) indicated that promoting blood circulation was the most advantageous in shortening lung rale disappearance time.

### Lung X-Ray Infiltrates Disappearance Time

The lung X-ray infiltrates disappearance time was assessed in four studies (392 patients) (Wang and Wu, 2016; Sun et al., 2017; Wang et al., 2018; Zhang, 2020b). Statistically significant differences between the groups were noted, which demonstrated that CHM combined with WM was superior [MD = −2.75, 95% CI (−3.33, −2.17), *p* < 0.00001, *I* ^*2*^ = 0%] (Figure 4B). In subgroup analysis (Table 2), TCM therapy of removing heat-phlegm and toxicity was more beneficial than invigorating Qi and consolidating superficies.

### Inflammation Index

Three studies (342 patients) (Zhong et al., 2017; Wang et al., 2018; Zhang, 2020b) mentioned the changes in tumor necrosis factor-α (TNF-α). The analysis result exhibited significant difference between two groups [MD = −5.49, 95% CI (−7.21, −3.77), *p* < 0.00001, *I* ^*2*^ = 58%] (Figure 4C), making it clear that CHM combined with WM was remarkably better than WM alone in reducing inflammation. In addition, the level of TNF-α seemed to be lower in the TCM therapy of removing heat-phlegm and toxicity than invigorating Qi and consolidating superficies (Table 2).

### Adverse Effects

One study (Wang et al., 2018) reported no adverse reactions during treatment. One (Chen, 2014) reported minor adverse reactions in two groups, but the number of cases was not shown. Seven studies (Lian, 2017; Sun et al., 2017; Tan and Yang, 2017; Wang, 2018; Yang, 2019; He et al., 2020a; He et al., 2020b) reported the number of adverse events and details were shown in Supplementary Table S2. All reactions were at a mild level, posing no influence on the therapy. No difference was represented between two groups [RR = 0.97, 95% CI (0.60, 1.57), *p* = 0.91; *I* ^*2*^ = 0%] (Figure 4D). The adverse reactions such as nausea, vomit, diarrhea, stomach ache, and rash were found in both groups, indicating those symptoms might be labeled in the direction of macrolide antibiotics and corticosteroids.

### Meta-Regression Analyses

Meta-regression analysis was attempted to explain the heterogeneity in antipyretic time and lung rale disappearance time. Neither type of WM (regression coefficient β = −0.677, SE = 0.487, *p* = 0.207) nor TCM therapy (regression coefficient β = 0.498, SE = 0.457, *p* = 0.312) had an association with antipyretic time. In terms of lung rale disappearance time, the type of WM (regression coefficient β = −1.027, SE = 0.341, *p* = 0.02) was more relevant than TCM therapy (regression coefficient β = 0.528, SE = 0.225, *p* = 0.051), indicating type of WM might be the source of heterogeneity.

### Publication Bias Analyses

**FIGURE 5:**
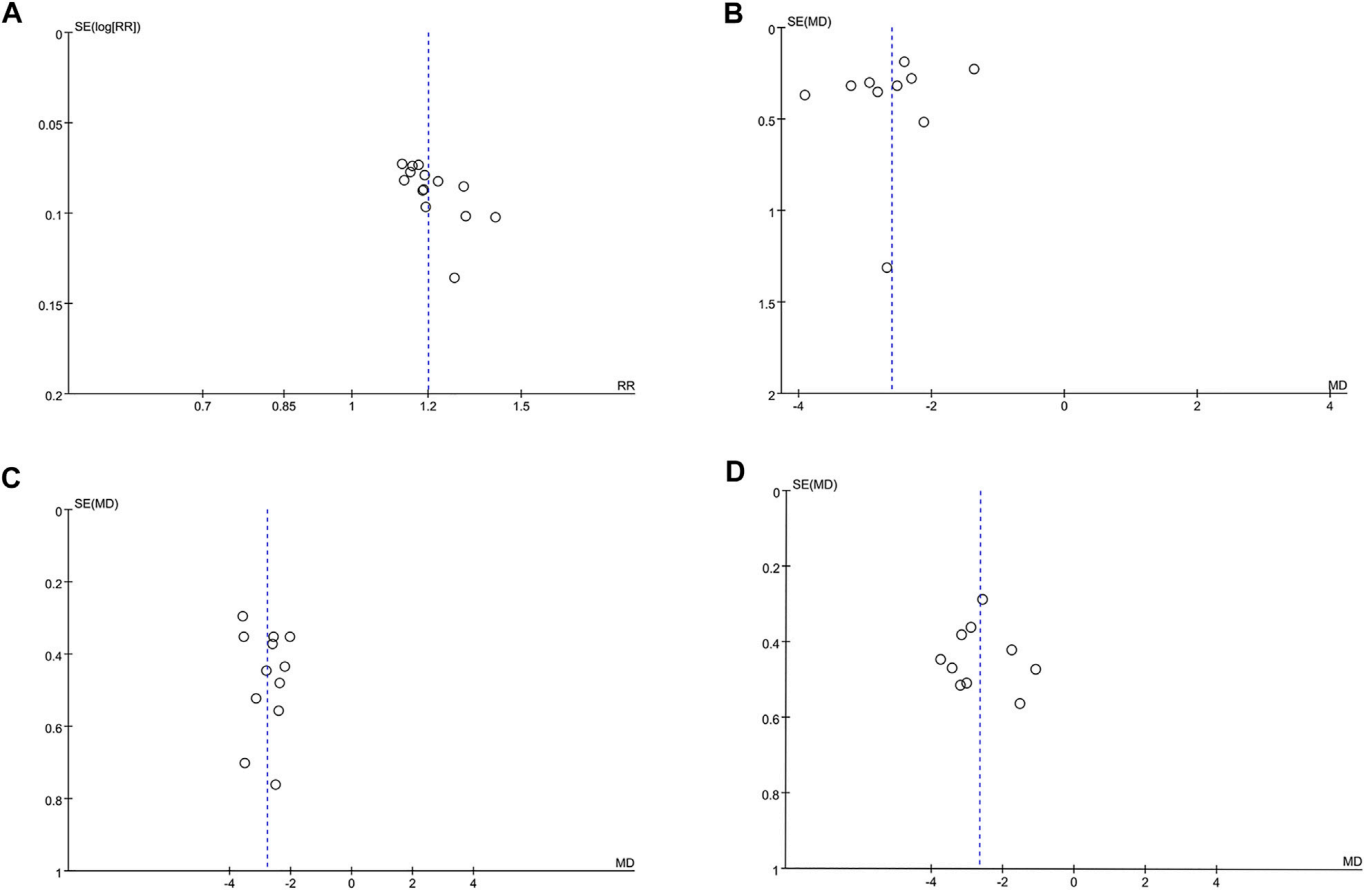
The funnel plots for assessing publication bias. **(A)** Clinical efficacy rate. **(B)** Antipyretic time. **(C)** Cough disappearance time. **(D)** Lung rale disappearance time.

Funnel plots and Egger’s test were performed for the assessment of publication bias. The funnel plot (Figure 5A) revealed a slight asymmetry and Egger’s test (t = 3.05, *p* = 0.01) indicated possible publication bias in clinical efficacy rate. To further confirm whether the bias had an impact on clinical efficacy rate, trim and fill method was performed. One study was added and the corrected RR was 1.493 [95% CI (1.349, 1.642)], remaining significant. Visual assessment of funnel plots (Figures 5B–D) and Egger’s test showed no publication bias for antipyretic time (t = −1.02, *p* = 0.338), cough disappearance time (t = 0.71, *p* = 0.495) and lung rale disappearance time (t = 0.30, *p* = 0.771).

### Sensitivity Analyses

All results showed good consistency whether in the fixed-effects or random-effects model. The overall results did not alter after deleting one study each time. For further verification, we implemented sensitivity analyses of clinical efficacy rate, antipyretic time, cough disappearance time and lung rale disappearance time by STATA 16.0. Supplementary Figure S1 indicated that the outcomes were not reversed by removing any study, which had relatively good stability.

When we deleted one study each time and reanalyzed the rest, we found the heterogeneity still existed except for TNF-α results of Zhong et al. (2017). The heterogeneity dropped from 58 to 0% after this study was excluded. Therefore, this RCT may be a source of considerable heterogeneity, in which azithromycin with intravenous injection was applied while trials of Wang et al. (2018) and Zhang (2020b) exerted azithromycin orally.

### Trial Sequential Analysis

**FIGURE 6:**
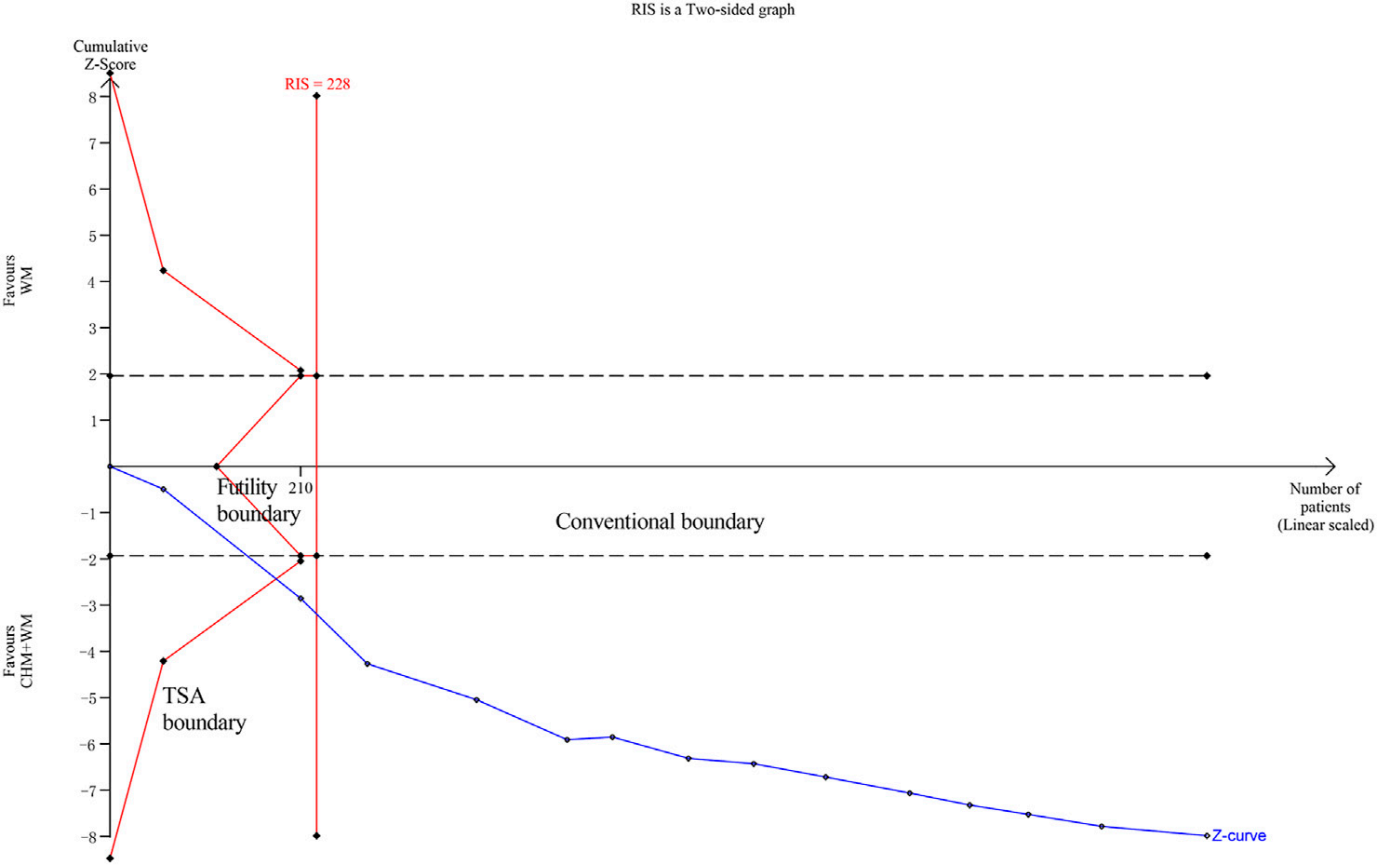
Trial sequential analysis of clinical efficacy rate.

The required information size (RIS), defined as the number of events or patients from the included studies necessary to accept or reject the statistical hypothesis (Wetterslev et al., 2009), is important to increase the quality of meta-analysis. Thus, We applied TSA boundaries to calculate the RIS and assess the robustness of our results. Relative risk reduction (RRR) was derived from the meta-analysis. In Figure 6, the Z-score curve (blue line) is found to cross the RIS boundary (vertical red line), TSA boundary (red polylines) and conventional boundary (dotted black lines), which indicated that the conclusion on clinical efficacy rate was robust with the existing evidence.

## Discussion

The main findings of our meta-analysis showed that the combination of CHM and WM could more significantly improve the clinical efficacy rate, relieve clinical symptoms (such as antipyretic time, cough disappearance time, lung rale disappearance time, lung X-ray infiltrates disappearance time) and decrease TNF-α level. Additionally, compared with WM, CHM therapies brought no increase in adverse reactions.

Since different regimens might result in the high heterogeneity. We performed subgroup analysis based on types of WM and different TCM therapies to obtain more reliable conclusion. The combination of CHM and WM showed obvious superiority whether compared with macrolide antibiotics or macrolide antibiotics and corticosteroids in the subgroup analysis. We also found that different TCM therapies had the similar clinical efficacy rate, but they targeted at different symptoms, which provided foundation for the application of CHM in RMPP.

**FIGURE 7:**
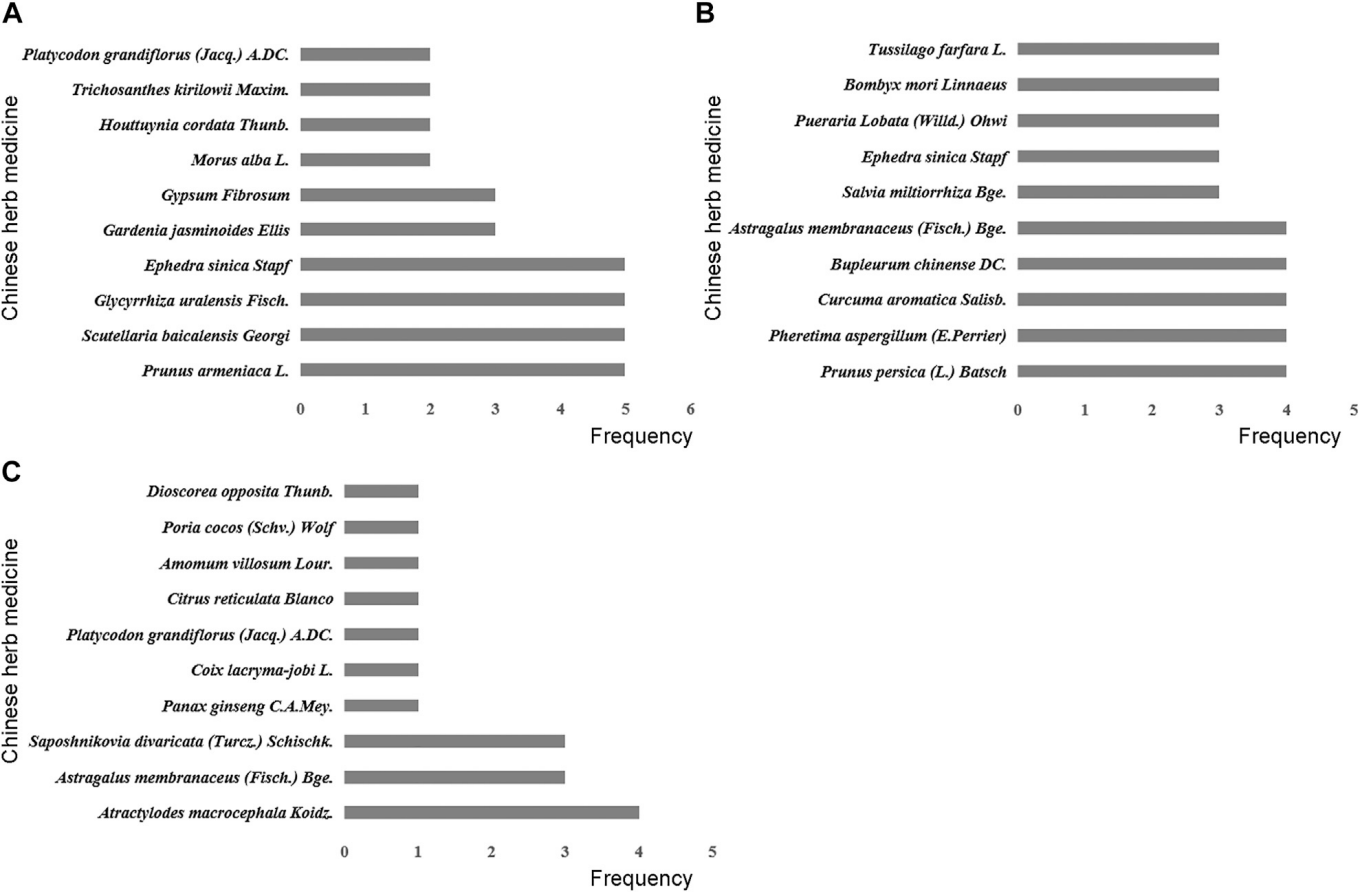
Frequency distribution of Chinese herb medicines in different traditional Chinese therapies. **(A)** Removing heat-phlegm and toxicity therapy. **(B)** Promoting blood circulation therapy. **(C)** Invigorating Qi and consolidating superficies therapy.

In the process of transforming MPP into RMPP, the monocytes and macrophages secreted large amounts of TNF-α, triggering a series of inflammatory reactions, immune disorders, thereby causing damage to organs. Some studies have demonstrated that TNF-α played an important role in the development of RMPP, which could be used as reference index for the prognosis judgment (Ding et al., 2018; Fan et al., 2019; Li et al., 2019a). As shown in our review, removing heat-phlegm and toxicity therapy had more advantages in shortening antipyretic time, cough disappearance time, lung X-ray infiltrates disappearance time and reducing TNF-α level. This might be due to anti-inflammation and anti-pathogen of Chinese herbs in the included RCTs, such as *Scutellaria baicalensis* Georgi., *Ephedra sinica* Stapf, *Prunus armeniaca* L., and *Glycyrrhiza uralensis* Fisch (Figure 7A). In TCM theory, they had the effects of clearing away heat and toxin, resolving phlegm and cough, indicating they could relieve post-infectious symptoms caused by *M. pneumoniae*. From the pharmacological point of view, baicalein, baicalin, wogonin, wogonoside and oroxylin A, the main active ingredients of *Scutellaria baicalensis* Georgi, were reported to inhibit the production of the inflammatory cytokines (such as interleukin-1β, interleukin-6, interleukin-8 and TNF-α), the molecular mechanisms of which included downregulation of toll-like receptors and inhibition of inflammation-associated pathways such as MAPK, Akt, NF-κB, and JAK-STAT (Liao et al., 2021). Meanwhile, baicalin have been proved to inhibit pathogen directly and reduce the expression of *M. pneumoniae* in a concentration-dependent manner (Meng et al., 2020). Additionally, Glycyrrhizic Acid contained in *Glycyrrhiza uralensis* Fisch. was thought to reduces cytokine secretion *via* blocking NF-κB activation and inhibiting the migration and infiltration of neutrophils, thereby attenuating inflammation in an acute lung injury mouse model (Lee et al., 2019).

The correlation of blood hypercoagulation with RMPP has been substantially demonstrated. It is attributed to the possible involvement of severe hypercoagulability and vascular endothelial cell injury, which results from an excessive inflammatory response, in lung injury (Huang et al., 2021). Based on TCM theory, “promoting blood circulation” was an important method for treating RMPP, which seemed to be better at relieving lung rale in our meta-analyses. The more frequently used Chinese herbs in this therapy contained *Prunus persica* (L.) Batsch and *Pheretima aspergillum* (E.Perrier) (Figure 7B), which have been documented as potent agents for the treatment of cough for nearly 2000 years. Modern pharmacological studies have found that *Prunus persica* (L.) Batsch and *Pheretima aspergillum* (E.Perrier) could ameliorate blood microcirculation through influencing hemorheological changes and platelet aggregation, which was vital for repairing tissue damage and improving respiratory function (Xu et al., 2015; Huang et al., 2018). Besides, an animal study suggested that the inflammatory response could be hindered by amygdalin, an active ingredient from *Prunus persica* (L.) Batsch, which is achieved through the downregulated expression of TNF-α, interleukin-6, interleukin-1β, and other pro-inflammatory cytokines (Lv et al., 2017).

T-lymphocyte subsets interact with each other and participate in the cellular immunity, so as to maintain the normal immune function of human body. Previous studies have reported T-lymphocyte subsets were closely related to the progression of RMPP, especially CD4^+^ T cells (Li et al., 2019b; Liu et al., 2020). In TCM, invigorating Qi and consolidating superficies herbs including *Atractylodes macrocephala* Koidz. and *Astragalus membranaceus* (Fisch.) Bge (Figure 7C). were expected to regulate immune system. For example, An animal study reported that *Atractylodes macrocephala* Koidz. water extract administered orally could increase the CD4^+^ T cell population in the spleen of mice stimulated with lipopolysaccharide (Kwak et al., 2018). According to an vitro study, polysaccharides derived from *Atractylodes macrocephala* Koidz. could exert immunoregulatory activities *via* regulating the proportions of CD8^+^ and CD4^+^ T cells (Sun et al., 2015). Astragaloside IV, which was known as the chief ingredient of *Astragalus membranaceus* (Fisch.) Bge., was proved to enhance immune cell proliferation and maintain homeostasis, and it could also reduce the expression of interleukin-4 to ameliorate airway inflammation (Huang et al., 2014).

Our review has presented consistent findings with a meta-analysis published in 2017, which demonstrated that CHM combined with WM could improve clinical efficacy compared with WM alone in children and adolescents with RMPP (Sheng and Huang, 2017). However, only eight RCTs (576 patients) with poor quality were included in the previous meta-analysis, and only clinical efficacy rate was evaluated, which made the conclusions unconvinced. Our research has unique strengths. First, we evaluated multiple outcome measures including some objective figures detected by machines to make our meta-analysis more comprehensive and credible. Second, dialectical diagnosis and treatment is the basic method of the TCM. Although children and adolescents suffer from the same disease, like RMPP, different syndrome types lead to different TCM therapies. Thus, we performed subgroup analysis based on different regimens, which could not only reduce heterogeneity but also compare the clinical efficacy of different TCM therapies in RMPP, providing good guidance for clinical practice.

Apart from efficacy, the safety of CHM in the treatment of RMPP is also an important issue worthy of consideration by clinicians. A total of nine studies in this meta-analysis reported safety, and no serious adverse events were observed. However, our results suggested that CHM had no advantage in alleviating the side effects, which was consistent with previous studies (Li, 2019). In addition, the description of adverse reactions in the instructions for Yupingfeng granule and Shedan Chuanbei oral liquid are “unclear” (Supplementary Table S4), indicating that there is no reliable clinical evidence to support their safety. Therefore, we considered the safety of CHM in the treatment of RMPP remained uncertain, which should be investigated in the further research.

### Limitations

This review is subject to some limitations. First, all included studies were performed in China and failed to describe the blinding method or allocation concealment. The poor quality of methodology might contribute to an exaggerated curative effect and decreased reliability of the evidence, thereby lowering the credibility and generalization of the results. Second, given the small sample size and limited number of studies in certain outcomes, the results might be insufficient to ensure a significant difference. Third, although sensitivity analysis and TSA demonstrated the reliability of our results, heterogeneity was observed in some results, such as antipyretic time and lung rale disappearance time. This might correlate with CHM intervention approaches, such as CHM composition, dose, and different forms administration. Thus, these results with substantial heterogeneity should be interpreted with caution. Last, drug safety is a key factor in clinical applications, but only nine RCTs described adverse events. Therefore, the safety of CHM in RMPP should be validated in future.

### Implication

The validity of the conclusion in meta-analysis was highly dependent on the quality of the RCTs included, thus more high-quality studies evaluating the efficacy and safety of CHM for RMPP are needed. Based on the above limitations, some recommendations are suggested for further studies: 1) Clinical trials should be registered in advance on clinical trials registry platform. 2) Methodological quality of future trials including randomization, allocation concealment, and blinding should be improved by using random number tables and opaque envelopes,etc. 3) The safety assessments of CHM need more attention. 4) Clinical trials of CHM should standardize syndrome differentiation and treatment, in order to investigate the clinical application of CHM better.

## Conclusion

In conclusion, CHM therapies together with WM seemed to be more beneficial for children and adolescents with RMPP in terms of relieving clinical symptoms, physical signs and reducing inflammation. However, due to the limitation of quality and the number of the included studies, we should take a cautious attitude toward these findings. Meanwhile, it is worth mentioning that the safety of CHM should be interpreted with caution. More RCTs with large samples and rigorous designs should be carried out to confirm the overall effect and further explore the optimal CHM therapies. In addition, more experimental studies are needed to provide evidence of CHM in treating RMPP at cellular and animal model levels and explore possible mechanisms.
