# Supplementary material for: Chinese Herbal Medicine for the Treatment of Children and Adolescents With Refractory Mycoplasma Pneumoniae Pneumonia: A Systematic Review and a Meta-Analysis
Source: Front Pharmacol. 2021 Jun 10;12:678631. doi: 10.3389/fphar.2021.678631 (PMC8222696; doi:10.3389/fphar.2021.678631)
Supplement: Supplementary file 1 [file DataSheet1.docx]

Supplementary Material

[Table S1 Search strategy 2](#_Toc52902052)

[Table S2 Characteristics of adverse events for included studies 5](#_Toc52902053)

[Table S3 Supplement Details respecting to the information of Self-made formulas 8](#_Toc52902054)

[Table S4 Supplement Details respecting to the information of Chinese patent medicines 1](#_Toc52902055)1

[File 1 PRISMA checklist. 1](#_Toc52902058)4

[File 2 A list of excluded studies by reading full text 1](#_Toc52902059)7

[FigureS1 Sensitivity analysis 2](#_Toc52902061)0

**Supplementary Table S1 Search strategy**

| **Databases** | **Search items** |
| --- | --- |
| Cochrane  Library | #1 (Medicine, Chinese Traditional) OR (Traditional Chinese Medicine) OR (Chung I HFTeh) OR (HFTeh, Chung I) OR (Traditional Medicine, Chinese) (Word variations have been searched)  #2 (Zhong Yi Xue) OR (Chinese Traditional Medicine) OR (Chinese Medicine, Traditional) OR (Traditional Tongue Diagnosis) OR (Tongue Diagnoses, Traditional) (Word variations have been searched)  #3 (Tongue Diagnosis, Traditional) OR (Traditional Tongue Diagnoses) OR (Traditional Tongue Assessment) OR (Tongue Assessment, Traditional) OR (Traditional Tongue Assessments) (Word variations have been searched)  #4 (Herbal Medicine) OR (Medicine, Herbal) OR (Herbalism) OR (Injections) OR (Injection) (Word variations have been searched)  #5 (Injectables) OR (Injectable) OR (decoction) OR (decoction) (Word variations have been searched)  #6 #1 OR #2 OR #3 OR #4 OR #5  #7 (refractory mycoplasma pneumoniae pneumonia) OR (RMPP) (Word variations have been searched)  #8 #6 AND #7  #9 MeSH descriptor: [Adolescent] explode all trees  #10 (Adolescents):ti,ab,kw OR (Adolescence):ti,ab,kw OR (Teens):ti,ab,kw OR (Teen):ti,ab,kw OR (Teenagers):ti,ab,kw OR (Teenager):ti,ab,kw OR (Youth):ti,ab,kw OR (Youths):ti,ab,kw OR (Adolescents, Female):ti,ab,kw OR (Adolescent, Female):ti,ab,kw OR (Female Adolescent):ti,ab,kw OR (Female Adolescents):ti,ab,kw OR (Adolescents, Male):ti,ab,kw OR (Adolescent, Male):ti,ab,kw OR (Male Adolescent):ti,ab,kw OR (Male Adolescents):ti,ab,kw  #11 MeSH descriptor: [Child] explode all trees  #12 (Children):ti,ab,kw  #13 #9 OR #10 OR #11 OR #12  #14 RCT  #15 #8 AND #13 AND #14 |
| Pubmed | ((refractory mycoplasma pneumoniae pneumonia) OR (RMPP)) AND (((((((((((((((((((((((((("Medicine, Chinese Traditional"[Mesh]) OR (Medicine, Chinese Traditional)) OR (Traditional Chinese Medicine)) OR (Traditional Medicine, Chinese)) OR (Zhong Yi Xue)) OR (Chinese Traditional Medicine)) OR (Chinese Medicine, Traditional)) OR (Traditional Tongue Diagnosis)) OR (Tongue Diagnoses, Traditional)) OR (Tongue Diagnosis, Traditional)) OR (Traditional Tongue Diagnoses)) OR (Traditional Tongue Assessment)) OR (Tongue Assessment, Traditional)) OR (Traditional Tongue Assessments)) OR ("Herbal Medicine"[Mesh])) OR (Herbal Medicine)) OR (Medicine, Herbal)) OR (Herbalism)) OR ("Injections"[Mesh])) OR (Injections)) OR (Injection)) OR (Injectables)) OR (Injectable)) OR (decoction)) OR (Drugs, Chinese Herbal) OR (Chinese patent medicine)) AND ((child[MeSH Terms]) OR (children[Title/Abstract])OR (("Adolescent"[MeSH Terms] OR "Adolescents"[Title/Abstract] OR "Adolescence Teens"[Title/Abstract] OR "Teen"[Title/Abstract] OR "Teenagers"[Title/Abstract] OR "Teenager"[Title/Abstract] OR "Youth"[Title/Abstract] OR "Youths"[Title/Abstract] OR "Adolescents, Female"[Title/Abstract] OR "Adolescent, Female"[Title/Abstract] OR "Female Adolescent"[Title/Abstract] OR "Female Adolescents"[Title/Abstract] OR "Adolescents, Male"[Title/Abstract] OR "Adolescent, Male"[Title/Abstract] OR "Male Adolescent"[Title/Abstract] OR "Male Adolescents"[Title/Abstract])) AND (randomized controlled trial[pt] OR controlled clinical trial[pt] OR randomized[tiab] OR placebo[tiab] OR clinical trials as topic[mesh:noexp] OR randomly[tiab] OR trial[ti]) NOT (animals [mh] NOT (humans [mh] AND animals[mh])) |
| EMBASE | #1 'refractory mycoplasma pneumoniae pneumonia'/exp OR 'refractory mycoplasma pneumoniae pneumonia'  #2 'medicine, chinese traditional'/exp OR 'medicine, chinese traditional'  #3 'traditional chinese medicine'/exp OR 'traditional chinese medicine'  #4 'chung i hFTeh'  #5 'hFTeh, chung i'  #6 'traditional medicine, chinese'  #7 'zhong yi xue'  #8 'chinese traditional medicine'  #9 'chinese medicine, traditional'  #10 'traditional tongue diagnosis'  #11 'tongue diagnoses, traditional'  #12 'tongue diagnosis, traditional'  #13 'traditional tongue diagnoses'  #14 'traditional tongue assessment'  #15 'tongue assessment, traditional'  #16 'traditional tongue assessments'  #17 'herbal medicine'/exp OR 'herbal medicine'  #18 'medicine, herbal'/exp OR 'medicine, herbal'  #19 'herbalism'  #20 'injections'/exp OR 'injections'  #21 'injection'/exp OR 'injection'  #22 'injectables'/exp OR 'injectables'  #23 'injectable'  #24 'decoction'/exp OR 'decoction'  #25 'chinese patent medicine'/exp OR 'chinese patent medicine'  #26 #2 OR #3 OR #4 OR #5 OR #6 OR #7 OR #8 OR #9 OR #10 OR #11 OR #12 OR #13 OR #14 OR #15 OR #16 OR #17 OR #18 OR #19 OR #20 OR #21 OR #22 OR #23 OR #24 OR #25  #27 'rmpp'  #28 #1 OR #27  #29 #26 AND #28  #30 'child'/exp  #31 'children':ab,ti  #32 'adolescent'/exp  #33 'adolescents':ab,ti OR 'adolescence':ab,ti OR 'teens':ab,ti OR 'teen':ab,ti OR 'teenagers':ab,ti OR 'teenager':ab,ti OR 'youth':ab,ti OR 'youths':ab,ti OR 'adolescents, female':ab,ti OR 'adolescent, female':ab,ti OR 'female adolescent':ab,ti OR 'female adolescents':ab,ti OR 'adolescents, male':ab,ti OR 'adolescent, male':ab,ti OR 'male adolescent':ab,ti OR 'male adolescents':ab,ti  #34 #30 OR #31 OR #32 OR #33  #35 'crossover procedure':de OR 'double-blind procedure':de OR 'randomized controlled trial':de OR 'single-blind procedure':de OR random*:de,ab,ti OR factorial*:de,ab,ti OR crossover*:de,ab,ti OR ((cross NEXT/1 over*):de,ab,ti) OR placebo*:de,ab,ti OR ((doubl* NEAR/1 blind*):de,ab,ti) OR ((singl* NEAR/1 blind*):de,ab,ti) OR assign*:de,ab,ti OR allocat*:de,ab,ti OR volunteer*:de,ab,ti  #36 #29 AND #34 AND #35 |
| CNKI | (SU = '难治性支原体肺炎' OR SU='RMPP' ) AND ( FT='中医' OR FT='中药' OR FT='中医药' OR FT='中西医' OR FT='汤' OR FT='片' OR FT='丸' OR FT='散' OR FT='胶囊' OR FT='颗粒' OR FT='水' OR FT='液' OR FT='合剂' OR FT='注射液') AND (SU='儿童' OR SU='小儿' OR SU='青少年' OR SU='学生') AND (FT='随机') |
| CBM | (("难治性支原体肺炎"[全部字段:智能] OR "RMPP"[核心字段:智能] )) AND(( "中医"[全部字段:智能] OR "中药"[全部字段:智能] OR ("中成药"[常用字段:智能] OR "中西医"[全部字段: 智能] OR"液"[全部字段:智能] OR "合剂"[全部字段:智能] OR "水"[全部字段:智能] OR "汤"[全部字段:智能]) OR "片"[全部字段:智能] OR "丸"[全部字段:智能] OR "散"[全部字段:智能] OR "胶囊"[全部字段:智能] OR "颗粒"[全部字段:智能] OR "注射液"[全部字段:智能]))AND ("儿童"[核心字段:智能] OR "小儿"[核心字段:智能] OR "青少年"[核心字段:智能] OR "学生"[核心字段:智能]) AND ("随机"[全部字段:智能]) |
| VIP | ((M=难治性支原体肺炎 OR RMPP ) OR (R=难治性支原体肺炎 OR RMPP )) AND ((U=中医 OR 中药 OR中成药 OR 中西医 OR 汤 OR 片 OR 丸 OR 散 OR 胶囊 OR 颗粒 OR 液 OR 水 OR 合剂 OR 注射液 ) OR (R=中医 OR 中药 OR中成药OR 中西医 OR 汤 OR 片 OR 丸 OR 散 OR 胶囊 OR 颗粒 OR 液 OR 水 OR 合剂 OR 注射液 )) AND ((M=儿童 OR 小儿OR 青少年) OR (R=儿童 OR 小儿OR 青少年OR 学生)) AND ((U=随机 ) OR (R=随机 )) |
| Wanfang  Data | 主题: (( "难治性支原体肺炎" OR "RMPP") AND ( "中医" OR "中药" OR "中成药" OR "中西医" OR "汤" OR "片" OR "丸" OR "散" OR "胶囊" OR "颗粒" OR "合剂" OR "水" OR "液" OR "注射液" ) AND ("小儿" OR "儿童" OR "青少年" OR "学生")) AND ("随机" ) |

**Supplementary Table S2 Characteristics of adverse events for included studies**

| **Study ID** | **Intervention** | | **Duration (weeks)** | **Adverse Effects** | | **Management for patients with adverse events (AEs)** |
| --- | --- | --- | --- | --- | --- | --- |
|  | **T** | **C** |  | **T** | **C** |  |
| Sun et al., 2017 | Yupingfeng granule + MA | MA | 2 | Six cases of gastrointestinal reactions. | Four cases of gastrointestinal reactions. | AEs were tolerable and did not affect treatment. |
| Meng, 2015 | Reduning injection + MA | MA | 1 | NR | NR | NR |
| Zhang, 2020b | Asarone injection + MA | MA | 2 | NR | NR | NR |
| Zhang, 2020a | Self-made formula + MA + CS | MA + CS | 2 | NR | NR | NR |
| Wang, 2018 | Self-made formula + MA + CS | MA + CS | 2 | Two cases of nausea and vomiting;three cases of diarrhea. | Three cases of nausea and vomiting. | AEs were tolerable and did not affect treatment. |
| Wang et al., 2018 | Yupingfeng granule + MA | MA | 2 | 0 | 0 | / |
| Wang and Wu, 2016 | Yupingfeng granule + MA + CS | MA + CS | 4 | NR | NR | NR |
| Tian, 2016 | Self-made formula + MA | MA | 1 | NR | NR | NR |
| Yuan et al.,  2016 | Self-made formula + MA | MA | 2 | NR | NR | NR |
| Tan and Yang, 2017 | Andrographolide injection + MA + CS | MA + CS | 1 | Four patients had nausea and vomiting;four patients had diarrhea. | Four patients had nausea and vomiting;three patients had diarrhea. | AEs were tolerable and did not affect treatment. |
| He et al., 2020a | Self-made formula + MA | MA | 2 | One patient had nausea;one patient had abdominal pain. | Three patients had nausea;two patients had abdominal pain;two patients had diarrhea. | AEs were tolerable and did not affect treatment. |
| He et al., 2020b | Self-made formula + MA + CS | MA + CS | 2 | Diarrhea in two patients;gastrointestinal reaction in two patients;rush in one patient. | Diarrhea in one patient;rush in three patients. | AEs were tolerable and did not affect treatment. |
| Lian, 2017 | Self-made formula + MA + CS | MA + CS | 2 | One patient had nausea and abdominal pain;two patients had rush. | Three patients had vomiting;one patient had granulocytosis. | AEs were tolerable and did not affect treatment. |
| Zhong et al., 2017 | Shedan Chuanbei oral liquid + MA | MA | 2 | NR | NR | NR |
| Qian, 2011 | Self-made formula + MA + CS | MA + CS | 2 | NR | NR | NR |
| Yang, 2019 | Self-made formula + MA + CS | MA + CS | 3 | Two patients had minor nausea and vomiting. | Three patients had minor nausea and vomiting;one patient had diarrhea. | AEs were tolerable and did not affect treatment. |
| Chen, 2014 | Self-made formula + MA | MA | 2 | Minor abdominal pain, diarrhea, nausea and vomiting.  (but number was unreported) | Minor abdominal pain, diarrhea, nausea and vomiting.  (but number was unreported) | AEs were tolerable and did not affect treatment. |

T, treatment; C, control; MA, macrolide antibiotics; CS, corticosteroids; NR, not reported; AEs, adverse events

**Supplementary Table S3 Supplement Details respecting to the information of Self-made formulas**

| **Study** | **Prescription name** | **Species, source, concentration** | **Form** | **Quality control reported?(Y/N)** | **Chemical analysis reported? (Y/N)** |
| --- | --- | --- | --- | --- | --- |
| Zhang, 2020a | Self-made formula | *Bupleurum chinense* DC*.* 5g; *Astragalus membranaceus* (Fisch.) Bge. 5g; *Salvia miltiorrhiza* Bge. 5g; *Prunus persica* (L.) Batsch 3g; *Glycyrrhiza uralensis* Fisch. 3g; *Citrus reticulata* Blanco 3g; *Pheretima aspergillum* (E.Perrier) 3g; *Curcuma aromatica* Salisb. 3g  [Miaoyu township CentralHospitals,  Wushan County, Chongqing] | Decoction | N | N |
| Wang, 2018 | Self-made formula | *Astragalus membranaceus* (Fisch.) Bge. 10g; *Salvia miltiorrhiza* Bge. 9g; *Bupleurum chinense* DC. 9g; *Ephedra sinica* Stapf 6g; *Pueraria Lobata* (Willd.) Ohwi 6g; *Prunus persica* (L.) Batsch 6g; *Bombyx mori* Linnaeus 6g; *Pheretima aspergillum* (E.Perrier) 6g; *Curcuma aromatica* Salisb. 6g; *Tussilago farfara* L. 6g  [Xuchang Central Hospital] | Decoction | N | N |
| Tian, 2016 | Self-made formula | *Astragalus membranaceus* (Fisch.) Bge. 12g; *Codonopsis pilosula* (Franch.) Nannf. 12g; *Pheretima aspergillum* (E.Perrier) 10g; *Curcuma aromatica* Salisb. 10g; *Prunus persica* (L.) Batsch 10g; *Bupleurum chinense* DC. 10g; *Aster tataricus* L.f. 10g; *Pueraria Lobata* (Willd.) Ohwi 10g; *Tussilago farfara* L. 10g; *Cryptotympana pustulata* Fabriciu 10g; *Bombyx mori* Linnaeus 10g; *Ephedra sinica* Stapf 6g  [Anping Maternal and Child Health Care Center,Hengshui City, Hebei Province] | Decoction | N | N |
| Yuan et al.,2016 | Self-made formula | *Morus alba* L. 10g; *Houttuynia cordata* Thunb. 10g; *Prunus armeniaca* L. 6g; *Scutellaria baicalensis* Georgi 6g; *Trichosanthes kirilowii* Maxim*.* 6g; *Citrus reticulata* Blanco 6g; *Platycodon grandiflorus* (Jacq.) A.DC. 6g; *Scrophularia ningpoensis* Hemsl. 6g; *Glycyrrhiza uralensis* Fisch. 3g; *Ephedra sinica* Stapf 2g  [Department of Traditional Chinese Medicine, Dongguan Wanjiang Hospital, Guangdong Province] | Decoction | N | N |
| He et al.,2020a | Self-made formula | Ages(3-5):*Coptis chinensis* Franch. 3g; *Scutellaria baicalensis* Georgi 3g; *Phellodendron amurense* Rupr*.* 5g; *Gardenia jasminoides* Ellis 6g; *Ephedra sinica* Stapf 3g; *Prunus armeniaca* L. 5g; *Glycyrrhiza uralensis* Fisch*.* 3g; *Gypsum Fibrosum* 10g  Ages(5-15):*Coptis chinensis* Franch. 6g; *Scutellaria baicalensis* Georgi 6g; *Phellodendron amurense* Rupr*.* 10g; *Gardenia jasminoides* Ellis 9g; *Ephedra sinica* Stapf 6g; *Prunus armeniaca* L. 10g; *Glycyrrhiza uralensis* Fisch*.* 6g; *Gypsum Fibrosum* 15g  [Cangzhou Central Hospital, Hebei Province] | Decoction | N | N |
| He et al.,2020b | Self-made formula | Ages(3-5):*Coptis chinensis* Franch. 3g; *Scutellaria baicalensis* Georgi 3g; *Phellodendron amurense* Rupr*.* 5g; *Gardenia jasminoides* Ellis 6g; *Ephedra sinica* Stapf 3g; *Prunus armeniaca* L. 5g; *Glycyrrhiza uralensis* Fisch*.* 3g; *Gypsum Fibrosum* 10g  Ages(5-15):*Coptis chinensis* Franch. 6g; *Scutellaria baicalensis* Georgi 6g; *Phellodendron amurense* Rupr*.* 10g; *Gardenia jasminoides* Ellis 9g; *Ephedra sinica* Stapf 6g; *Prunus armeniaca* L. 10g; *Glycyrrhiza uralensis* Fisch*.* 6g; *Gypsum Fibrosum* 15g  [Cangzhou Central Hospital, Hebei Province] | Decoction | N | N |
| Lian, 2017 | Self-made formula | *Ephedra sinica* Stapf 9g; *Prunus armeniaca* L. 12g; *Gypsum Fibrosum* 18g; *Salvia miltiorrhiza* Bge. 9g; *Ligusticum chuanxiong* Hort. 9g; *Scutellaria baicalensis* Georgi 12g; *Paeonia lactiflora* Pall. 9g; *Glycyrrhiza uralensis* Fisch. 9g  [Zhengzhou First People's Hospital, Henan Province] | Decoction | N | N |
| Qian, 2011 | Self-made formula | *Ephedra sinica* Stapf 2g; *Prunus armeniaca* L. 6g; *Morus alba* L. 10g; *Scutellaria baicalensis* Georgi 6g; *Houttuynia cordata* Thunb. 10g; *Trichosanthes kirilowii* Maxim. 6g; *Citrus reticulata* Blanco 6g; *Platycodon grandiflorus* (Jacq.) A.DC. 6g; *Scrophularia ningpoensis* Hemsl. 6g; *Glycyrrhiza uralensis* Fisch. 3g  [The 452nd Hospital of the PLA, 38th Branch of the Chengdu Military Area Command] | Decoction | N | N |
| Yang, 2019 | Self-made formula | *Panax ginseng* C.A.Mey. 5g; *Atractylodes macrocephala* Koidz. 10g; *Coix lacryma-jobi* L. 10g; *Platycodon grandiflorus* (Jacq.) A.DC. 6g; *Citrus reticulata* Blanco 5g; *Amomum villosum* Lour. 6g; *Poria cocos* (Schv.) Wolf 10g; *Dolichos lablab* L. 10g; *Dioscorea opposita* Thunb. 10g; *Nelumbo nucifera* Gaertn.10g; *Glycyrrhiza uralensis* Fisch. 5g  [Hengyang Hospital of Traditional Chinese Medicine] | Decoction | N | N |
| Chen, 2014 | Self-made formula | *Salvia miltiorrhiza* Bge. 12g; *Astragalus membranaceus* (Fisch.) Bge. 12g; *Prunus persica* (L.) Batsch 10g; *Curcuma aromatica* Salisb. 10g; *Pheretima aspergillum* (E.Perrier) 10g; *Bombyx mori* Linnaeus 10g; *Pueraria Lobata* (Willd.) Ohwi 10g; *Cryptotympana pustulata* Fabriciu 10g; *Bupleurum chinense* DC. 10g; *Tussilago farfara* L. 10g; *Aster tataricus* L.f. 10g; *Ephedra sinica* Stapf 6g  [Nankang First People's Hospital, Jiangxi Province] | Decoction | N | N |

**Supplementary Table S4 Supplement Details respecting to the information of Chinese patent medicines**

| **Study** | **Formulation** | **Source** | **Species,**  **concentration** | **Botancical plant names** | **Quality control reported?(Y/N)** | **Chemical**  **analysis**  **reported? (Y/N)** | **Chemical composition standard** | **Adverse drug**  **reactions** |
| --- | --- | --- | --- | --- | --- | --- | --- | --- |
| Sun et al., 2017；Wang et al., 2018；  Wang and Wu, 2016 | Yupingfeng granule | Guangdong Global Pharmaceutical Group co., Ltd. | Astragali Radix 600g;  Atractylodis Macrocephalae  Rhizoma 200g;  Saposhnikoviae Radix 200g | Root of *Astragalus membranaceus* (Fisch.) Bge*.*; rhizome of *Atractylodes macrocephala* Koidz*.*; root of *Saposhnikovia divaricata* (Turcz.) Schischk. | Y-National Food and Drug Administration National Drug Standards.  Standard number: Z10930036 | N | Astragaloside IV (C_41_H_68_O_14_ ) ＞ 0.7mg/g | Unclear |
| Meng, 2015 | Reduning injection | Jiangsu Kangyuan Pharmaceutical Group co., Ltd. | Artemisiae Annuae  Herba;  Lonicerae Japonicae  Flos;  Gardznize Fructus. | Dry aerial part of *Artemisia annua* L*.*; dried flower of *Lonicera japonica* Thunb.; dried fruit of *Gardenia jasminoides* J.Ellis | Y-National Food and Drug Administration National Drug Standards.  Standard number: Z20050217 | N | Gardenoside (C_17_H_24_O_10_):7.2-12.6 mg/ml;  Secoxyloganin  (C_17_H_24_O_11_):0.8-2.1 mg/ml;  Chlorogenic acid  (C_16_H_18_O_9_):4.6-7.8 mg/ml; Neochlorogenic acid (C_16_H_18_O_9_):1.8-3.7 mg/ml; Cryptochlorogenic acid (C_16_H_18_O_9_): 1.7-3.6 mg/ml | 1.Some patients have dizziness, chest tightness, dry mouth, diarrhea, nausea and vomiting;2.There are systemic redness, pruritus or rash and other allergic reactions in occasionally. |
| Zhang, 2020b | Asarone injection | Hunan Dinuo Pharmaceutical Group co., Ltd. | α-Asarone | Rhizome of *Acorus tatarinowii* Schott | Y- National Food and Drug Administration National Drug Standards.  Standard number: H20058790 | N | α-Asarone (C_12_H_16_O_3_): 90% - 110% | Minor adverse effects, such as dry mouth, dizziness, nausea, stomach discomfort, palpitations and constipation, may occur in a small number of people, and shock is rare. |
| Tan and Yang, 2017 | Yanhuning injection | Chongqing Yaoyou Pharmaceutical Group co., Ltd. | Potassium Sodium  Dehydroandro grapholide  Succinate | Dry aerial part of Andrographis paniculata (Burm.f.) Nees | Y- National Food and Drug Administration National Drug Standards.  Standard number: H50021640 | N | Potassium Sodium Dehydroandrographolide Succinate (C_28_H_34_KNaO_10_·H_2_0) : 90% - 110% | 1. Allergic reactions. It can be manifested as rash, pruritus, maculopapule, severe or even dyspnea, edema, anaphylactic shock, mostly in the first drug use; 2. Gastrointestinal reactions. Nausea, vomiting, diarrhea, and liver function impairment have also been reported. 3. Blood system reaction. Visible leucopenia, thrombocytopenia, purpura. 4. Thermogenic sample reaction. Chills, high fever, even dizziness, chest tightness, palpitations, tachycardia, blood pressure drop. |
| Zhong et al.,2017 | Shedan Chuanbei oral liquid | Guangdong Yililuoding Pharmaceutical Group co., Ltd. | Serpentis fellis 10g;  Fritiliariae Cirrhosae Bulbus 75g | *Fel Serpentis*; rhizome of *Fritillaria cirrhosa* D.Don | Y**-**Z44023603 issued by National Food and Drug Administration National Drug Standards. | N | Taurocholate hydrate (C_26_H_45_NO_7_S ) ＞0.07 mg/ml | Unclear |

**Supplementary File 1 The PRISMA checklist of this meta-analysis**

| **Section/topic** | **#** | **Checklist item** | **Reported on page #** |
| --- | --- | --- | --- |
| **TITLE** | | |  |
| Title | 1 | Identify the report as a systematic review, meta-analysis, or both. | 1 |
| **ABSTRACT** | | |  |
| Structured summary | 2 | Provide a structured summary including, as applicable: background; objectives; data sources; study eligibility criteria, participants, and interventions; study appraisal and synthesis methods; results; limitations; conclusions and implications of key findings; systematic review registration number. | 1-2 |
| **INTRODUCTION** | | |  |
| Rationale | 3 | Describe the rationale for the review in the context of what is already known. | 2-3 |
| Objectives | 4 | Provide an explicit statement of questions being addressed with reference to participants, interventions, comparisons, outcomes, and study design (PICOS). | 3 |
| **METHODS** | | |  |
| Protocol and registration | 5 | Indicate if a review protocol exists, if and where it can be accessed (e.g., Web address), and, if available, provide registration information including registration number. | 3  CRD42020218609 |
| Eligibility criteria | 6 | Specify study characteristics (e.g., PICOS, length of follow-up) and report characteristics (e.g., years considered, language, publication status) used as criteria for eligibility, giving rationale. | 4 |
| Information sources | 7 | Describe all information sources (e.g., databases with dates of coverage, contact with study authors to identify additional studies) in the search and date last searched. | 3 |
| Search | 8 | Present full electronic search strategy for at least one database, including any limits used, such that it could be repeated. | 3; Supplement Table S1 |
| Study selection | 9 | State the process for selecting studies (i.e., screening, eligibility, included in systematic review, and, if applicable, included in the meta-analysis). | 4 |
| Data collection process | 10 | Describe method of data extraction from reports (e.g., piloted forms, independently, in duplicate) and any processes for obtaining and confirming data from investigators. | 4 |
| Data items | 11 | List and define all variables for which data were sought (e.g., PICOS, funding sources) and any assumptions and simplifications made. | 4 |
| Risk of bias in individual studies | 12 | Describe methods used for assessing risk of bias of individual studies (including specification of whether this was done at the study or outcome level), and how this information is to be used in any data synthesis. | 4 |
| Summary measures | 13 | State the principal summary measures (e.g., risk ratio, difference in means). | 4-5 |
| Synthesis of results | 14 | Describe the methods of handling data and combining results of studies, if done, including measures of consistency (e.g., I^2^) for each meta-analysis. | 5 |

| **Section/topic** | **#** | **Checklist item** | **Reported on page #** |
| --- | --- | --- | --- |
| Risk of bias across studies | 15 | Specify any assessment of risk of bias that may affect the cumulative evidence (e.g., publication bias, selective reporting within studies). | 4 |
| Additional analyses | 16 | Describe methods of additional analyses (e.g., sensitivity or subgroup analyses, meta-regression), if done, indicating which were pre-specified. | 5 |
| **RESULTS** | | |  |
| Study selection | 17 | Give numbers of studies screened, assessed for eligibility, and included in the review, with reasons for exclusions at each stage, ideally with a flow diagram. | 5; Figure1 |
| Study characteristics | 18 | For each study, present characteristics for which data were extracted (e.g., study size, PICOS, follow-up period) and provide the citations. | 5-6 |
| Risk of bias within studies | 19 | Present data on risk of bias of each study and, if available, any outcome level assessment (see item 12). | 6;  Figure2 |
| Results of individual studies | 20 | For all outcomes considered (benefits or harms), present, for each study: (a) simple summary data for each intervention group (b) effect estimates and confidence intervals, ideally with a forest plot. | 6-8;  Figure3-4 |
| Synthesis of results | 21 | Present results of each meta-analysis done, including confidence intervals and measures of consistency. | 8-9 |
| Risk of bias across studies | 22 | Present results of any assessment of risk of bias across studies (see Item 15). | 8  Figure5 |
| Additional analysis | 23 | Give results of additional analyses, if done (e.g., sensitivity or subgroup analyses, meta-regression [see Item 16]). | 8-9 |
| **DISCUSSION** | | |  |
| Summary of evidence | 24 | Summarize the main findings including the strength of evidence for each main outcome; consider their relevance to key groups (e.g., healthcare providers, users, and policy makers). | 9-11 |
| Limitations | 25 | Discuss limitations at study and outcome level (e.g., risk of bias), and at review-level (e.g., incomplete retrieval of identified research, reporting bias). | 12 |
| Conclusions | 26 | Provide a general interpretation of the results in the context of other evidence, and implications for future research. | 12 |
| **FUNDING** | | |  |
| Funding | 27 | Describe sources of funding for the systematic review and other support (e.g., supply of data); role of funders for the systematic review. | 13 |

*From:*  Moher D, Liberati A, Tetzlaff J, Altman DG, The PRISMA Group (2009). Preferred Reporting Items for Systematic Reviews and Meta-Analyses: The PRISMA Statement. PLoS Med 6(6): e1000097. doi:10.1371/journal.pmed1000097

For more information, visit: **www.prisma-statement.org**.

**Supplementary File 2** **A list of excluded studies by reading full text**

**Non-randomized controlled trials (n=13)**

1. Cheng, K., and Liang, H.Y. (2019). Clinical study on the treatment of refractory mycoplasma pneumonia in children with ziwanbaihua powder and azithromycin. *J Shanxi Univ Chin Med* 42(02), 99-100+114. doi: 10.13424/j.cnki.jsctcm.2019.02.029.
2. Deng, M.H. (2015). Treating refractory Mycoplasma pneumoniae pneumonia with Maxin Shigan Decoction. *Clin J Chin Med* 7(07), 90-91. doi: 10.3969/j.issn.1674-7860.2015.7.050.
3. Li, N. (2019a). Clinical study on the treatment of refractory mycoplasma pneumonia (damp-heat syndrome) in children by clearing damp tongluo method and its effect on the expression of IL-10 and TNF-α in RAW264.7 cells. *Shandong Univ Chin Med*.
4. Li, S.F. (2019b). Clinical Observation on the Treatment of Infantile Refractory Mycoplasma Pneumoniae Pneumonia. *J Guangming Chin Med* 34(17), 2712-2713. doi: 10.3969/j.issn.1003-8914.2019.17.048.
5. Li, X.B., and Tong, X.H. (2017). Analysis of the effect of integrated traditional Chinese and western medicine on children with refractory mycoplasma pneumonia. *J Mod Med Health Res Electronic* 1(07), 59.
6. Liu, D.D. (2015). Combination of Traditional Chinese and Western Medicine in Treating Refractory Mycoplasma Pneumonia in Children. *Med Health Care* (4), 25-26.
7. Liu, X.H., Cui, H., and Han, W.J. (2012). Summary of Professor Li Gui's experience in using integration of traditional and western medicine to treat pediatric persistent refractory mycoplasma pneumoniae pneumonia. *J Capit Univ Med Sci* 33(03), 311-314. doi: 10.3969/j.issn.1006-7795.2012.03.006.
8. Liu, X.S., Yang, H.F., Ban, D.D., and Zhang, H.W. (2018). Analysis of the efficacy of azithromycin combined with Qingkailing oral liquid in the treatment of refractory mycoplasma pneumonia in children. *Mod Diagn Treat* 29(22), 3609-3611. doi: 10.3969/j.issn.1001-8174.2018.22.016.
9. Liu, X.X., Wang, Y., Chen, P., Xu, J.L., and Liu, Y.J. (2019). Clinical Obervation of In-vitro Cultured Calculus Bovis Combined with Methylprednisone Sodium Succinate Injection in the Treatment of Refractory Mycoplasma Pneumonia. *Chin J Integr Tradit West Med* 39(08), 960-964. doi: 10.7661/j.cjim.20190111.035.
10. Sun, G.M., Xu, A.H., Dong, H.Y., Qi, X.H., and Wang, Y.L. (2004). Observation on Treatment of Refractory Mycoplasma Pneumoniae Pneumonia by Combination of Chinese and Western Medicines. *J Pract Tradit Chin Med* (10), 566-567. doi: 10.3969/j.issn.1004-2814.2004.10.035.
11. Wang, Q., Zhu, S., Zhao, Y.H., and Wang, H.L. (2015). Treatment of Intractable Pediatric Mycoplasma Pneumonia by Qingfei Huoxue Recipe Combined Azithromycin: a Random Parallel Control Study. *Chin J Integr Tradit West Med* 35(05), 545-548. doi: 10.7661/CJIM.2015.05.0545.
12. Zhang, W.H. (2014). Clinical Observation on the Treatment of Infantile Refractory Mycoplasma Pneumoniae Pneumonia. *Asia-Pacific Tradit Med* 10(08), 60-61.
13. Zhao, J.L. (2013). Clinical analysis of persistent and refractory mycoplasma pneumonia in children. *J Jilin Med* 34(11), 2067-2068. doi: 10.3969/j.issn.1004-0412.2013.11.058.

**Inappropriate interventions (n=13)**

1. Chen, L. (2015). Clinical analysis of refractory mycoplasma pneumoniae pneumonia in children. *J Chin Mod Pharm Appl* 9(16), 146-147. doi: 10.14164/j.cnki.cn11-5581/r.2015.16.102.

2. Hong, L.J., Wang, C.L., Hong, B.S., and Jiang, P.Y. (2010). Clinical observation on the effect of the treatment combining traditional Chinese with western medicine for intractable mycoplasma pneumonia. *Chin Integr Tradit West Med Pediatrics* 2(06), 517-519. doi: 10.3969/j.issn.1674-3865.2010.06.018.

3. Li, Y.L., Song, T., Sui, Y., and Xiao, Z.Z. (2016). Safety Observation on the Treatment of Infantile Refractory Mycoplasma Pneumoniae Pneumonia. *J Integr Tradit West Med World* 11(01), 77-79. doi: 10.13935/j.cnki.sjzx.160123.

4. Liu, J.H. (2014). Clinical analysis of 38 children with refractory mycoplasma pneumoniae pneumonia. *J Chin Foreign Med Research* (9), 131-132.

5. Lv, Q. (2019). Treatment of refractory mycoplasma pneumonia with traditional Chinese medicine combined with acupoint application and its effect on children's serum IgE. *Yunnan J Tradit Chin Med Mater Medica* 40(06), 74-75. doi: 10.3969/j.issn.1007-2349.2019.06.032.

6. Ma, Y.X., and Song, W.K. (2020). Clinical Study on Treating Refractory Mycoplasma Pneumoniae Pneumonia with Xiaoerchiqiaokeli and Small Dose Hormone in Children. *J Asia-Pacific Trad Med* 16(3), 146-148. doi: 10.11954/ytctyy.202003048.

7. Qiu, Z.J., Chen, L.X., and Li, J. (2015). Therapy of Integrated Medicine in the Treatment of Refractory Mycoplasma Pneumoniae in Children for 42 Cases. *Chin Med Mod Dist Edu* 13(24), 60-61. doi: 10.3969/j.issn.1672-2779.2015.24.031.

8. Wang, J. (2012). Curative Effect Observation on the Treatment of 24 Cases of Infantile Refractory Mycoplasma Pneumoniae Pneumonia. *Chin J Integr Tradit West Med Pediatrics* 4(06), 536-537. doi: 10.3969/j.issn.1674-3865.2012.06.026.

9. Wang, S.L., Hou, J.H., Zhang, Y., Guo, Y.R., and Li, F. (2020). Clinical Efficacy of Modified Weijingtang and Maxing Shigan Tang Combined with Half-dose Hormones on Refractory Mycoplcasma Pneumoniae Pneumonia in Children Caused by Toxic Heat Closing Lung. *J Chin Exp Trad Med Formulae* 26(10), 69-74. doi: 10.13422/j.cnki.syfjx.20201021.

10. Zhang, Q., and Wang, C.H. (2016). Improvements of immune function and clinical symptoms of pidotimod combined with Yanhuning for children with refractory mycoplasma pneumoniae pneumonia. *Chi Pri Med* 23(20), 3175-3179. doi: 10.3760/cma.j.issn.1008-6706.2016.20.035.

11. Zhang, S.M. (2013). Clinical effect of integrated traditional Chinese and western medicine in the treatment of refractory mycoplasma pneumonia in children. *Med Info* 26(10), 414. doi: 10.3969/j.issn.1006-1959.2013.10.520.

12. Zhou, B., Li, Y., and Yuan, J. (2010). Clinical analysis of intractable mycoplasma pneumonia for 38 children. *Chin J Integr Tradit West Med Pediatrics* 02(6), 540-541. doi: 10.3969/j.issn.1674-3865.2010.06.030.

13. Zhou, Y.L., Wang, Q.L., Wang, J.X., Zhang, J.J., and Li, J. (2019). Observation on the Safety and Efficacy of Integrated Traditional Chinese and Western Medicine in the Treatment of Infantile Refractory Mycoplasma Pneumoniae Pneumonia. *J Chin rural health* 11(4), 4-5. doi: 10.3969/j.issn.1674-361X.2019.04.005.

**Outcomes not meeting the inclusion criteria (n=8)**

1. Guo, X.J., and Wei, G.L. (2016). Analysis of the effect of Qingrejiedu Recipe on improving C-reactive protein in children with refractory mycoplasma pneumonia. *Anti-Infection Pharm* 13(03), 703-705. doi: 10.13493/j.issn.1672-7878.2016.03-089.
2. Wang, C.L., Hong, L.J., Zhao, C.B., Chen, C.H., and Wang, F. (2017). Clinical Observation of Jinbei Kangzhi Syrup in the Treatment of Refractory Mycoplasma Pneumoniae Pneumonia. *China's Naturopathy* 25(03), 38. doi: 10.3969/j.issn.1007-5798.2017.03.033.
3. Yang, H.R., Liu, J.H., and Shuai, J.F. (2014). Effect of Qingre Jiedu Formula on T-Lymph Subsets of Children with Refractory Mycoplasma Pneumonia. *J Hebei Tradit Chin Med Pharm* 29(01), 6-8.
4. Yang, H.R., Liu, J.H., Zhao, X.M., Shuai, J.F., Niu, B., and Lu, S.K. (2014b). Effect of Qingrejiedu Decoction on C-reactive Protein in Children with Refractory Mycoplasma Pneumonia. *Hebei Med* 20(12), 2031-2033. doi: 10.3969/j.issn.1006-6233.2014.12.036.
5. Yang, H.R., Liu, J.H., Zhao, X.M., Shuai, J.F., Niu, B., and Lu, S.K. (2014b). Effect of Qingre Jiedu Formula on Living Mycoplasma in Children with Refractory Mycoplasma Pneumonia. *J Hebei Tradit Chin Med Pharm* 29(02), 10-11+16.
6. Yang, H.R., Liu, J.H., Zhao, X.M., Shuai, J.F., Niu, B., and Lu, S.K. (2018). Effect of Qingrejiedu Recipe on the amount of mycoplasma in the alveolar lavage fluid of children with mycoplasma pneumonia. *J Hebei Tradit Chin Med Pharm* 33(03), 21-23.
7. Zhu, H.Y. (2017). Effect of Qingrejiedu prescription on the Level of IFN-γ IL-4 and IL-6 of Children with Refractory Mycoplasma Pneumonia. *Hebei Med* 23(07), 1132-1135. doi: 10.3969/j.issn.1006-6233.2017.07.023.
8. Zhou, Y.Y. (2018). Effect of Qingfei Huoxue Zhike Decoction on TCM Syndrome Score and Inflammatory Reaction of Refractory Mycoplasma Pneumonia in Children. *J Shanxi Med* 47(17), 2097-2098. doi: 10.3969/j.issn.0253-9926.2018.17.045.

**Lack of adequate drug information (n=2)**

1. Cai, X.H., and Xie, C. (2014). Clinical study on the treatment of refractory mycoplasma pneumonia with pidotimod dispersible tablet combined with Bailing capsule. *J Chin Mod Drug App* 8(11), 141-143.
2. Wang, Q.Q., Su, W.D., Zhang, W.W., and Lan, Y.L. (2018). The efficacy of asarone injection combined with bronchoalveolar lavage for refractory Mycoplasma pneumonia in Children. *Chin Gen Pract* 16(07), 1137-1140. doi: 10.16766/j.cnki.issn.1674-4152.000313.

**Without access to full text (n=2)**

1. Liu, L.L. (2015). Therapeutic effect analysis of heat-clearing and nourishing lung therapy on refractory mycoplasma pneumonia in children. *Qingdao Jiaozhou Central Hospital*.
2. Ge, L. (2018). The immunomodulatory effect of Huaiqihuang Granules on children with severe or refractory Mycoplasma pneumoniae pneumonia. *Dalian Med Univ*.

**Supplementary Figure S1 Sensitivity analysis**


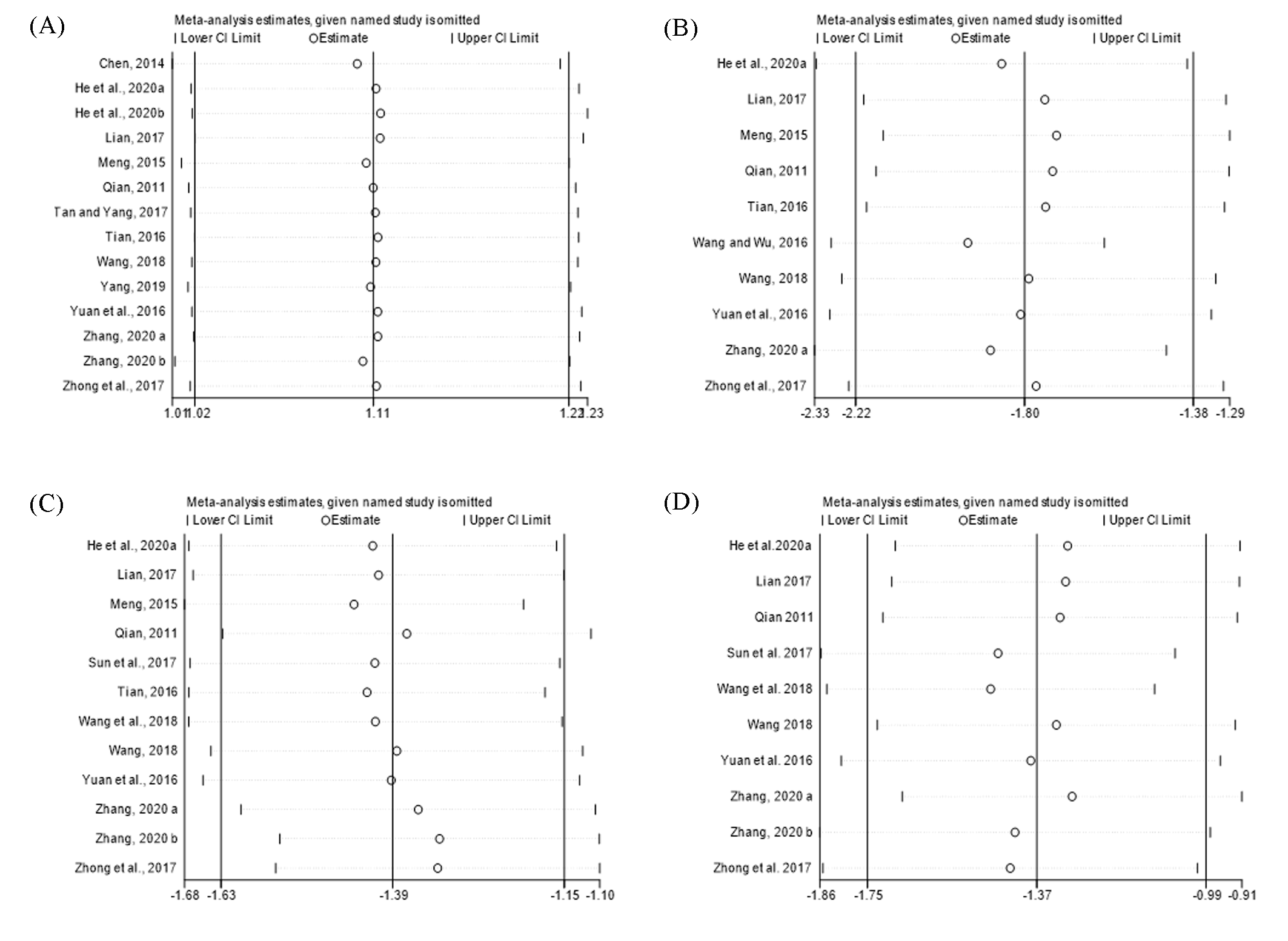


1. Clinical efficacy rate. (B) Antipyretic time. (C) Cough disappearance time. (D) Lung rale disappearance time
